# Bacterial Toxins and the Nervous System: Neurotoxins and Multipotential Toxins Interacting with Neuronal Cells

**DOI:** 10.3390/toxins2040683

**Published:** 2010-04-15

**Authors:** Michel R. Popoff, Bernard Poulain

**Affiliations:** 1 Institut Pasteur, Bactéries anaérobies et Toxines, 25 rue du Dr Roux, F-757254, Paris cedex 15, France; 2 Neurotransmission et Sécrétion Neuroendocrine, CNRS UPR 2356 IFR 37 - Neurosciences, Centre de Neurochimie, 5, rue Blaise Pascal, F-67084 STRASBOURG cedex, France; Email: poulain@neurochem.u-strasbg.fr

**Keywords:** toxin, neurotoxin, enterotoxin, nervous system, actin cytoskeleton, small gtpases, neurotransmitter

## Abstract

Toxins are potent molecules used by various bacteria to interact with a host organism. Some of them specifically act on neuronal cells (clostridial neurotoxins) leading to characteristics neurological affections. But many other toxins are multifunctional and recognize a wider range of cell types including neuronal cells. Various enterotoxins interact with the enteric nervous system, for example by stimulating afferent neurons or inducing neurotransmitter release from enterochromaffin cells which result either in vomiting, in amplification of the diarrhea, or in intestinal inflammation process. Other toxins can pass the blood brain barrier and directly act on specific neurons.

## 1. Introduction

**Figure 1:**
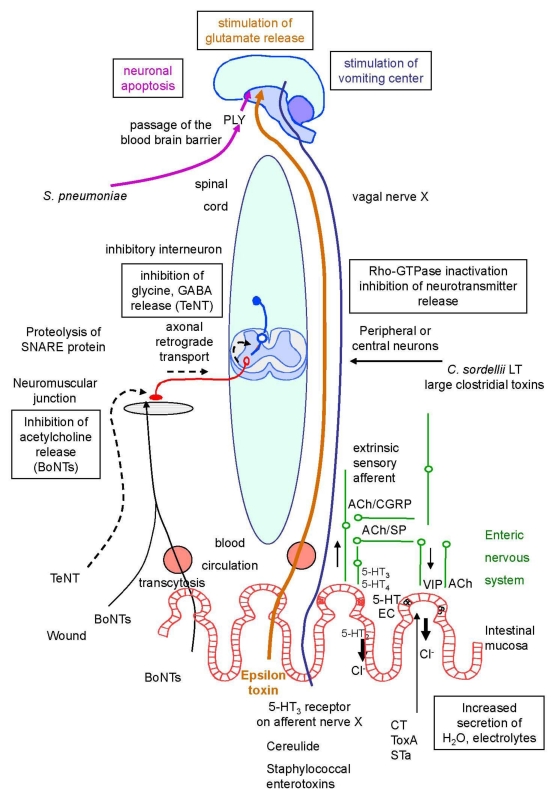
Schematic representation of bacterial toxins active on the nervous system.

Pathogenic bacteria use various strategies to interact with target tissues and cells of the host organism. Among them, toxin production constitutes an efficient way to alter specific functions of target cells. Toxins can act locally or at a distance from the infectious site and are responsible for severe diseases in man and animals. While some toxins, called cytotoxins, cause disruption of all cell types, thus permitting the pathogen access to nutrients, other toxins are only active on specific cells such as enterotoxins active on epithelial intestinal cells and neurotoxins targeting neuronal cells. This is achieved by the recognition of specific cell surface receptor(s) and/or specific intracellular target(s). When bound to the receptor, toxins can unleash their toxic program at the cell membrane by interfering with signal transduction pathways, pore formation, or enzymatic activities towards membrane compounds. In contrast, other toxins enter the cytosol, recognize, and modify specific intracellular targets. According to the nature of the target and the type of modification, intracellular active toxins cause a dramatic alteration of cellular functions such as protein synthesis, cell homeostasis, cell cycle progression, vesicular traffic, and actin cytoskeletal rearrangements. The nervous system which is diffused through all the organism is one of the main target of bacterial toxins. If neurotoxins, like clostridial neurotoxins, exclusively interact with neuronal cells from the central or peripheral nervous system inducing specific neurological symptoms, other toxins recognize a broader range of cell types including neuronal cells. For example, cytotoxins can trigger necrosis or apoptosis in various cell types as well as in neuronal cells. Thereby various toxins, in addition to their specific activity on some cell types, affect certain neuronal cells, directly or indirectly, leading to neurological symptoms associated with typical clinical signs resulting from the other affected cells (Table 1, and Figure 1). This review is focused on the various modes of interaction with neuronal cells of bacterial neurotoxins and other toxins affecting the nervous system.

## 2. The Cellular and Molecular Mechanisms Involved in Neuroexocytosis: An Overview

### 2.1. An overview of neurotransmission

Transfer of information or command between neurons, or neurons and target cells (muscle fibers, endocrine cells, *etc*.) is most often chemical in nature and occurs at highly specialized contact sites termed *synapses*. Here, the release of neurotransmitter molecules by the presynaptic elements enables activation of receptors localized on the postsynaptic target. Neurotransmitter molecules are comprised of small organic molecules as acetylcholine (ACh), catecholamines like dopamine or noradrenaline, serotonin (5-HT), glutamate, gamma-aminobutyric acid (GABA), glycine, adenosine-triphosphate (ATP), and numerous peptides such as vaso-intestinal peptide (VIP), substance P (SP), and calcitonin gene-related protein (CGRP). Released transmitter substance(s) can activate ligand-gated ionic channels or metabotropic receptors, thus mediating either transmembrane ionic fluxes or activation of intracellular signaling pathway(s). For example, in the central nervous system, depending on the ion species flowing through the channel, activation of ligand-gated ionic channels can cause depolarization (*i.e.*, excitation) or hyperpolarization (*i.e.*, inhibition) of the postsynaptic plasma membrane, respectively, and the excitation/inhibition net balance determines eventual initiation of action potentials propagated in the neuron until the next synapses. The evoked endplate potential at muscle fibers following stimulation of the motor nerve (and subsequent ACh release) is a depolarization that may reach the threshold for initiating muscle action potential, which itself propagates along muscle fiber ultimately triggering its contraction. In the enteric nervous system, release of neurotransmitter molecules (as VIP) by mucosal nerve endings directly contacting the enterocytes, or indirectly *via* the activation of entero-chrommaffin cells releasing 5-HT, can lead to activation of metabotropic receptors, intracellular activation of the adenylate cyclase and downstream cAMP-dependent pathways, resulting in an active co-transport of ions species (Na^+^, K^+^, Cl^−^) and water efflux for osmotic compensation in the intestine lumen (viz the molecular mechanisms of diarrhoea [5]). 

**Table 1:** Bacterial neurotoxins and other toxins interacting with the nervous system.

Toxin	Bacteria	Structure	Target neuronal cell	Receptor	Activity	Effects	**MLD (μg/kg)^1^**
**Toxins inhibiting the neuroexocytosis**							
Botulinum neurotoxins	*C. botulinum*	single chain protein (150 kDa)	motoneurons	gangliosides (GD_1b_, GT_1b_)	proteolysis of SNARE proteins (VAMP, SNAP25, syntaxin)	inhibiton of acetylcholine release (flaccid paralysis)	0.0003
	*C. baratii*			synaptotagmin, SV2			
	*C. butyricum*						
Tetanus neurotoxin	*C. tetani*	single chain protein (150 kDa)	inhibitory interneurons	gangliosides (GD_1b_, GT_1b_)	proteolysis of SNARE protein (VAMP)	inhibition of neurotransmitter release (GABA, glycine) (spastic paralysis)	0.001
				GPI-anchored protein			
Lethal toxin	*C. sordellii*	single chain protein (250 kDa)	potentially all neurons	unknown	inactivation of Rho and Ras-GTPases (glucosylation)	inhibition of neurotransmitter release	0.1
Toxin B	*C. difficile*	single chain protein (250 kDa)	potentially all neurons	unknown	inactivation of Rho-GTPases (glucosylation)	inhibition of neurotransmitter release	32
Pneumolysin	*S. pneumoniae*	single chain protein (53 kDa)	hippocampal neurons	cholesterol	pore-forming activity	neuronal apoptosis (meningitis)	
Enterotoxin	*C. perfringens*	single chain protein (36 kDa)	enterocyte neurons		pore-forming activity		80
**Toxins stimulating neurosecretion**							
Epsilon toxin	*C. perfringens*	single chain protein (36 kDa)	hippocampal neurons	unknown	pore-forming activity	stimulation of glutamate release (excitation)	0.1
Cholera toxin	*V. cholerae*	AB5 structure	enterochrompaffin cells and enteric neurons	ganglioside GM_1_	inactivation of Gsα and activation of adenylate cyclase	5-HT release (diarrhea)	250
Heat labile enterotoxin	*E. coli*	AB5 structure	enterochromaffin cells and enteric neurons	ganglioside GM_1_	inactivation of Gsα and activation of adenylate cyclase	5-HT release (diarrhea)	250
Toxin A	*C. difficile*	single chain protein (300 kDa)	enterocytes enteric neurons	membrane glycoprotein	inactivation of Rho-GTPases, other mechanism?	release of inflammatory mediators and neuropeptides (diarrhea)	0.35
Heat stable enterotoxin	*E. coli*	short peptide (2–5 kDa)	enterocyte enteric neurons?	guanylate cyclase	GMP_c_ increase,other mechanism?	stimulation of enteric nervous system(diarrhea)	
Staphylococcal enterotoxins	*S. aureus*	single chain protein (25–30 kDa)	enterochromaffin cells, vagal nerve	histocompatibility complex class II molecules	superantigen other mechanism?	5-HT release stimulation of 5-HT_3_ receptor (emesis)	20 (monkey)
Cereulide	*B. cereus*	cyclic dodecadepsipeptide (1.2 kDa)	vagal nerve	5HT_3_ receptor	K^+^ ionophore	stimulation of 5-HT_3_ receptor (emesis)	

^1^Mouse lethal doses per kg of body weight according to [1,2,3,4].

In nerve terminals, transmitter molecules are stored into at least two different classes of secretory organelles: the small lucent synaptic vesicles, and large dense core vesicles or granules. The small lucent vesicles, approximately 50 nm in diameter, contain small organic molecules and are formed by either budding from the early endosome or recycling of empty vesicles [6,7]. The large dense core vesicles are analogous to the secretory granules present in endocrine and exocrine cells and have a biogenesis different from that of the synaptic vesicles. Similar to many cargo vesicles, they are formed by vesicle budding from the trans-Golgi system, followed by homotypic fusion to form larger vesicles. Usually they mediate release of peptide transmitter or certain catecholamines whose synthesis depends on the presence in the granule lumen of enzymes (as the dopamine-beta-hydroxylase). Small synaptic vesicles and certain large dense core vesicles are equipped with a vacuolar-type ATPase that creates a proton gradient which drives specific vesicular transporters, thereby allowing active uptake and storage of neurotransmitter molecules inside these vesicles. Regardless the secretory organelle size, large or small, the release of its content into the extracellular space implicates a fusion of its membrane with the plasmalemma, which then allows passive diffusion of its content into the surrounding medium. This exocytotic process is triggered by a rise in the cytosolic concentration of Ca^++^, in response either to the arrival of a propagated action potential (*i.e.*, at the neuron nerve endings) or following activation of ionotropic or/and metabotropic receptors located on the plasma membrane of secretory cells [8].

### 2.2. Mechanisms of exocytosis and SNAREs

More than 100 proteins are implicated in Ca^++^-dependent exocytosis. However, only a dozen of them participate in the core machinery required for transmitter release, while the others serve regulatory roles [9,10]. Interestingly, several proteins of the release machinery are targeted and disabled by various bacterial toxins. A large body of evidence indicates that the synaptic vesicles do not move freely within the nerve ending cytosol, and regulation of their interaction with actin-based cytoskeleton allows fine tuning of their movements [11,12]. At the fusion site, they bind to the scaffold proteins associated with a special presynaptic matrix termed “active zone” [12]. When tethered to the fusion site, the synaptic vesicles are not yet fusogenic and must acquire fusion competence. A key step in this process is the pairing and assembly of a fusion particle comprised of VAMP (vesicle-associated membrane protein, also termed synaptobrevin), SNAP-25, and syntaxin, on the inner face of the plasma membrane (reviewed by [9,10]). These three proteins are also designed as the SNARE proteins (soluble *N*-ethylmaleimide-sensitive factor attachment protein receptors). Munc-18 protein is the fourth essential protein partner, it participates in SNARE complexe formation and binds to it (reviewed by [9,10]).The SNARE proteins or closely related isoforms are also involved in the fusion of large dense core vesicle or secretory granules. Assembly of the three SNAREs, and possibly munc-18, triggers hemifusion of vesicles and plasma membrane. Termination of fusion is triggered by a rise in intracellular Ca^++^ concentration, which may result from either Ca^++^ influx through voltage-gated channels (e.g., at nerve terminals), Ca^++^ release from intracellular stores, or both (e.g., at many neuroendocrine cells) [13,14]. The principal Ca^++ ^sensor for triggering fusion is synaptotagmin, which is an integral synaptic vesicle protein interacting with SNAREs [10,15,16,17]. Synaptotagmin is equipped with two “C2-domains”, which acquire high affinity for membrane lipids upon binding to Ca^++^ ions but in a pretty high intracellular concentration (10–100 µM). Thus synaptotagmin is likely sensing the Ca^++^ changes due to activation of voltage-dependent calcium channels. Its Ca^++^-dependent interaction with plasma membrane leads to changes in membrane curvature and destabilization of the lipids permitting the fusion [18]. Several SNARE/synaptotagmin complexes (possibly 4 to 6) must act synergistically to allow fusion of a synaptic vesicle (reviewed by [9,10]). Recently, a role similar to that of synaptotagmin has been assigned to the Doc2 protein, given the high affinity of Doc2 for Ca^++^. Thus, Doc2 is likely intervening in spontaneous exocytosis at nerve endings and secretory cells, which is triggered/regulated by minute changes in the resting intracellular Ca^++^ concentration [19].

### 2.3. Actin cytoskeleton and small GTPases in exocytotic mechanisms

Many other proteins play key roles in synaptic vesicle trafficking and priming of tethered synaptic vesicles. Inside nerve terminals, vesicles traffic along actin filaments and this implicates molecular motors like myosins II or V, and small GTPases of the Rab family (Rab3 and others) [20,21]. Reorganization of the actin cytoskeleton is coupled to Ca^++^-regulated exocytosis in endocrine cells. However, this is far from being clear in neurons [11,22,23]. The role of actin in exocytosis seems to consist in governing the vesicle-granule trafficking towards release site [22,23]. Organization of the actin-based cytoskeleton is controlled by several proteins, including several small GTPases like Rho proteins [24] and ADPribosylation factor (ARF). Like most small GTPases, ARF and Rho proteins cycle between GDP-bound (inactive), and GTP-bound (active) states, thereby acting as signal transducers that respond to upstream signals. Thus, they activate downstream effector molecules which carry out their biological functions. Rho proteins (Rho, Rac, Cdc42) are widely-expressed monomeric GTPases. Their translocation to specific membrane domains enables intervention of distinct biological functions, including: (1) regulation of actin cytoskeletal dynamics; (2) cell cycle progression; (3) gene transcription; (4) membrane transport; and 5) exo-/endo-cytosis [23,24]. In chromaffin and PC12 cells, RhoA associates with secretory granules, whereas Rac1 and Cdc42 are found in the subplasmalemmal region [23]. Activation of phosphatidylinositol 4-kinase by RhoA promotes the formation of granule-associated actin filaments and/or stabilize the subplasmalemmal actin barrier [23]. In chromaffin cells, Cdc42 and Rac1 control actin polymerization and secretion [25,26]. In neurons, Rac1 is associated with synaptic vesicles and plasma membrane [27]. Rac1 is involved in a post-docking step of neuronal exocytosis during which it controls in an all-or-none manner the functionality of release sites [27,28], possibly *via* regulation of phospholipase D (PLD) activity [29]. Additional pathways converging on PLD1 implicates ARF6 GTPases [30]. Ral-GTPase is abundant in nerve terminals and associates with synaptic vesicles [31]. This molecule apparently plays a key role in neurotransmitter release by regulating the pool size of readily releasable synaptic vesicles [32]. Ral has been implicated in regulating PLD activity too [33]. Downstream from ARF6, Ral, Rho, Rac, and Cdc42, PLD produces PA. PLD is possibly activated by these GTPases upon docking of synaptic vesicles, or secretory granules, at the release sites. PLD activation is an important event for exocytosis in neurons and many secretory cell types [34,35,36,37]. PLD-production of phosphatidic acid (PA) may either signal attachment of some proteins of the fusion machinery to the fusion site or play a role in vesicle fusion. Indeed, PA is a cone-shaped lipid whose local accumulation, and possibly destabilization of the lipids at the fusion site [35,36,37] may promote negative curvation of the inner (cytoplasmic) plasma membrane leaflet [38].

## 3. Toxins Inhibiting the Neuroexocytosis

### 3.1. Toxins which specifically impair the SNARE exocytosis mechanism: Clostridial neurotoxins

*Clostridium* *botulinum* and *Clostridium* *tetani* secrete very potent neurotoxins, which are responsible for neurological disorders in humans and animals, botulism and tetanus, respectively. Several recent reviews detail the structure and mode of action of neurotoxins [39,40,41,42,43,44,45].

*C.* *tetani* forms a homogeneous bacterial species which produces only one type of tetanus toxin (TeNT), whereas botulinum neurotoxin (BoNT)-producing strains are heterogeneous. *C.* *botulinum* is divided into 4 groups, which on the basis of phenotypic and genotypic parameters, correspond to different species. In addition, some strains of other species, such as *Clostridium* *butyricum* and *Clostridium* *baratii*, can produce a related BoNT type E and F, respectively. Seven BoNT toxinotypes (A, B, C, D, E, F, and G) are distinguished according to their antigenic properties. Each toxinotype is now divided into subtypes based on BoNT sequence variations, which can impact antibody binding and neutralization or affinity as well as catalytic efficiency for their substrate [46,47,48,49,50,51].

BoNTs are associated with non-toxic proteins (ANTPs) to form large complexes. ANTPs encompass a non-toxic and non-hemagglutinin component (NTNH) and several hemagglutinin components (HA34, HA17 and HA70 in *C.* *botulinum* A) or OrfX components [44,52]. Recent model and composition of BoNT complexes have been proposed [53,54]. The function of ANTPs is still unclear; they could protect the neurotoxin from the acidic pH of the stomach and from digestive proteases.

#### 3.1.1. Structure

BoNTs and TeNT share a common structure. They are synthesized as a precursor protein (about 150 kDa), which is inactive or weakly active. The precursor which does not contain signal peptide, is released from the bacteria possibly by a yet misunderstood cell-wall exfoliation mechanism [55]. The precursor is proteolytically activated in the extra-bacterial medium either by *Clostridium* proteases or by exogenous proteases such as digestive proteases in the intestinal content. The active neurotoxin consists of a light chain (L, about 50 kDa) and a heavy chain (H, about 100 kDa), which remain linked by a disulfide bridge. The structure of BoNTs shows three distinct domains: L-chain containing α-helices and β-strands and including the catalytic zinc binding motif, the N-terminal part of the H-chain forming two unusually long and twisted α-helices, and the C-terminal part of the H-chain consisting of two distinct subdomains (H_CN_ and H_CC_) involved in the recognition of the receptor. While the three domains are arranged in a linear manner in BoNT/A and BoNT/B, both the catalytic domain and the binding domain are on the same side of the translocation domain in BoNT/E. This domain organization in BoNT/E might facilitate a rapid translocation process [56,57,58,59,60,61,62,63,64,65].

The overall sequence identity at the amino acid level beween BoNTs and TeNT ranges from 34 to 97%. Several domains are highly conserved which account for the common mode of action of these toxins. Thereby, the central domains of L chains are related in all the clostridial neurotoxins and contain the consensus sequence (His-Glu-X-X-His) characteristic of zinc-metalloprotease active site. The half N-terminal domain of the H-chains is also highly conserved, and it is involved in the translocation of the L-chain into the cytosol. Thus, a similar mechanism of internalization of the intracellular active domain into target cells is shared by all the clostridial neurotoxins. In contrast, the half C-terminal parts of H-chain, mainly the H_cc_ subdomains, are the most divergent [44,52]. This accounts for the different receptors recognized by the clostridial neurotoxins (see below).

#### 3.1.2. Mode of action

Although BoNTs and TeNT use different routes, they display a similar intracellular mechanism of action. BoNTs enter by oral route or are produced directly in the intestine subsequently to a *C.* *botulinum* intestinal colonization and then undergo a transcytosis across the digestive mucosa [66,67,68,69,70,71]. After diffusion into the extracellular fluid and blood stream circulation, BoNTs target motoneuron endings. In contrast, TeNT is formed in wounds colonized by *C.* *tetani*. TeNT diffuses in the extracellular fluid and can target all types of nervous endings (sensory, adrenergic neurons and motoneurons), but it is mainly retrogradelly transported through the motoneurons (see below) [72,73,74].

Each type of BoNT and TeNT recognizes specific receptors on demyelinated terminal nerve endings, mainly through the H_CC_ subdomain. BoNT/A, /C, /E, /F exploit the three isoforms of the vesicle protein SV2 as specific receptors, while BoNT/B and /G bind to synaptotagmin I or II [75,76,77,78,79,80,81]. The GPI-anchored membrane protein Thy-1 has been proposed to act as a TeNT receptor [82,83], but this has not been confirmed. Ganglioside-binding sites have been characterized in the H_CC_ subdomain. Interestingly, TeNT exhibits two carbohydrate-binding sites, whereas BoNT/A and BoNT/B show only one [63,84,85,86]. Accordingly, TeNT can bind simultaneously to two gangliosides [87]. BoNT/C and BoNT/D interact with gangliosides (GD_1b_, GT_1b_) and phosphatidylethanolamine, respectively by their H_CC_ subdomain [88]. The role of H_CN _subdomain, which may interact with phosphatidylinositol phosphates [89], is still unclear. Overall, whatever the considered clostridial neurotoxin, the identified protein receptors are not neurospecific and are expressed on several cell types including intestinal crypt epithelial cells in the intestine [69]. Distribution of the gangliosides recognized by BoNTs differs from that of the protein receptors. Thus, the high affinity of BoNTs and TeNT for presynaptic membranes probably results from multiple and synergistic interactions with the ganglioside and protein parts of receptor, and binding to gangliosides which induces conformational changes in the Hc domain, probably facilitates subsequent binding to protein receptor [90,91]. Co-presence of the *ad hoc* ganglioside(s) and protein receptors likely facilitates the identification of cell subset targeted by TeNT or BoNTs at very low concentrations encountered in the physiological medium during the disease. At higher concentrations, binding to the protein receptor is likely sufficient for mediating toxin binding. Indeed the number of cell types affected by these toxins expands with increasing toxin concentrations. Therefore, BoNTs can target numerous neurons but not all, as well as non-neuronal cells at high concentrations, inhibiting the release of various compounds (Table 2).

**Table 2:** Clostridial neurotoxins block the release of many different neurotransmitters and other molecules in neuronal and in some non-neuronal cells. However, the release of certain neurotransmitters is resistant to BoNTs, either the target cells do not express the specific receptor(s) for BoNT entry or the intracellular target (SNARE protein) do not contain BoNT cleavage site.

Neurotransmitter	Model system	References
**Blockage of neurotransmitter and other molecule release by BoNTs in neuronal cells**		
Acetylcholine (ACh)	Skeletal muscular junction	[92]
	Torpedo electric organ	[93]
	Aplysia, CNS	[94]
Glutamate	Brain synaptosome	[95]
	Hind paw/mass spectrometry	[96]
	Cultured rat cerebellar neurons/radioassay	[97]
	Cultured rat cerebellar neurons/enzymatic assay	[98]
Aspartate	Brain synaptosomes	[99]
Gamma aminobutyric acid (GABA)	Brain synaptosomes	[99,100]
Glycine	Spinal cord neurons (culture)	[101]
Dopamine	Brain synaptosomes	[100,102]
Adrenalin		
Noradrenalin		
Serotonin (or 5-HT)	Brain synaptosomes	[103]
ATP corelease with ACh	Torpedo synaptosomes	[104]
	Rat bladder urothelium	[105,106]
	Guinea pig stellate neurons	[107]
Nicotinamide adenine dinucleotide (NAD)	Canine mesenteric artery	[108]
	Human urinary bladder detrusor muscle	[109]
**Effects on other neuropeptides**		
Substance P (SP)	Inhibition of KCl evoked SP release	
Calcitonin gene-related peptide (CGRP)	Inhibition of release in cultured dorsal root ganglia (DRG) neurons	[110,111]
	Inhibition of release in cultured rat bigeninal nerve cells	[112]
	Rat bladder afferent neurons	[113]
	Upregulation and increase of CGRP	[114,115,116,117]
**Neurotransmitter release resistant to BoNTs**		
Vaso intestinal peptide (VIP), CGRP	Periglandular innervation of sweat glands	[118]
Neuropeptide Y	Vasoconstrictor neurons afferent to vena cava and uterine artery from guinea pig	[119]
SP	Capsain evoked release from cultured DRG	[110]
Nitric oxide (NO) Ach	Non vesicular fraction of 5-HT evoked ACh release at bronchiolar smooth muscle (ACh release by epithelial cells ?)	[119,120,121]
GABA	Cultured inhibitory hippocampal interneurons (BoNT-resistant SNAP25 related isoform ?)	[122]
**Blockage of the process release in non-neuronal cells by BoNTs at high concentrations (≥100 nM)**		
Catecholamines	Chromaffin cells	[123,124,125]
ATP, glutamate	Glial cells: astrocytes or Schwann cells	[126,127,128]
Insulin	Pancreatic beta-cells	[129]
Store-mediated Ca^++^ entry	Exocrine pancreas cells	[130]
Store-mediated Ca^++^ entry	Platelets	[131]
**Blockage of neurotransmitter by TeNT**		
Glycine	Cat, rat spinal cord	[132,133]
	Murine spinal cord cell cultures (complete blockage of evoked and spontaneous release)	[134]
GABA	Rat brain	[135]
	Pig cerebrocortical synaptosomes	[99]
	Rat hippocampal slices	[136,137,138]
	Cerebellar cell cultures	[139]
Glutamate	Murine spinal cord cell cultures (partial blockage of evoked release and increase in spontaneous release)	[134]
	Rat brain	[140]
	Cerebellar neuronal cells	[141]
	Pig cerebrocortical synaptosomes	[99,142]
	Cultured hippocampal neurons (blockage of AMPA receptor insertion *via* SNARE-dependent exocytosis)	[143,144]
	Synaptosomes	[142]
Aspartate	Pig cerebrocortical synaptosomes	[99]
	Brain synaptosomes	[138]
	Brain slices	[145]
	Synaptosomes (no inhibition of evoked aspartate release)	[142]
Catecholamines	Cultured brain neurons	[146]
	Chromaffin cells	[147,148,149,150,151]
	Synaptosomes	[152,153]
	Rat brain	
Serotonin	Rat brain	[152,153]
	Synaptosomes	[154]
	Synaqptosomes (inhibition of serotonin uptake)	[155,156,157,158]
Acetylcholine	Chromaffin PC12 cells	[159,160]
	Synaptosomes	[161,162]
	Aplysia californica neurons (intraneural injection)	[163]
Met-enkephalin	Pig cerebrocortical synaptosomes	[99]
**Blockage of exocytosis in non neuronal cells by TeNT**		
Glutamate	Astrocytes	[164]
Transferrin receptor	CHO cell (cleavage of cellubrevin)	[165]

Neurotoxin bound to its receptor is internalized by receptor-mediated endocytosis. An essential difference between both types of neurotoxins is that BoNTs are directly endocytosed in clathrin-coated vesicles, which, when acidified, trigger the translocation of the L chain into the cytosol. Therefore, BoNT L chain is delivered in the peripheral nervous system, to neuromuscular junctions where it blocks the release of acetylcholine leading to a flaccid paralysis. In contrast, TeNT enters different endocytic vesicles, which are not acidified. The vesicles retrogradely transport the toxin in a microtubule-dependent manner to the cell body of neurons in the spinal cord. Like nerve growth factors, TeNT is transported by tubulo-vesicular organelles characterized by the presence of neurotrophin receptor such as p75^NTR^[166,167,168,169,170,171]. The C-terminal fragment of TeNT drives the retrograde transport of the toxin, and can be used to transport heterologous protein in the same way [172,173]. Then, TeNT carries out a transynaptic migration and reaches the target neurons, which are inhibitory interneurons involved in the regulation of the motoneurons. TeNT enters target inhibitory interneurons *via* vesicles that are acidified thus permitting the delivery of the L chain into the cytosol, where it inhibits the regulated release of glycine and GABA. Acidification of the vesicle lumen triggers a conformational change of the neurotoxin and subsequent translocation of the L chain into the cytosol. H chains form tetramers and insert into lipid membranes, thus forming cation selective channels permeable to small molecules (<700 Da). The mechanism of translocation is not completely understood. The N-terminal part of H chain mediates the translocation of L chain into the cytosol at acidic endosomal pH by modifying the electrostatic interactions with the phospholipids without detectable conformational changes. In addition, the disulfide bond between the two chains has a crucial role in the translocation process [174,175,176,177]. Then, the L chain refolds in the neutral pH of the cytosol. Cytosolic translocation factors such as β-COPI are possibly involved in this mechanism, as it has been found for diphtheria toxin [41,43,45,178]. 

**Figure 2:**
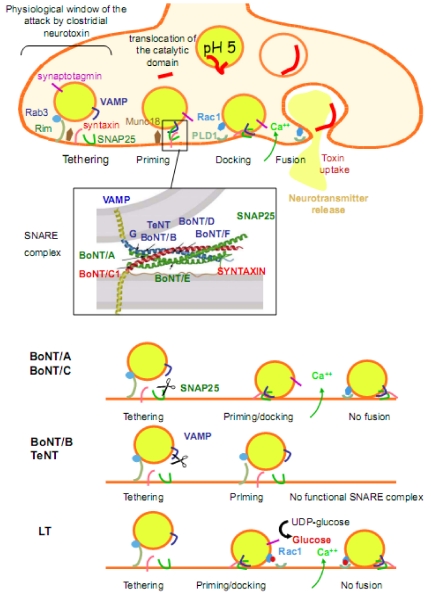
Schematic representation of the molecular mechanism of evoked neuroexocytosis and of toxin-dependent inhibition of neurotransmitter release. Clostridial neurotoxins are assumed to enter neuronal cells *via* synaptic vesicle recycling or by classical receptor-dependent endocytosis like *C. sordellii* LT. Acidification of the endosomal lumen triggers the translocation of the catalytic domain (L chain of clostridial neurotoxins or N-terminal domain of LT) into the cytosol. Synaptic vesicles loaded with neurotransmitter move from a reserve zone to the “active zone” in a close proximity to the release sites through an actin filament-dependent manner. Synaptic vesicles association with release sites is first driven by interaction of the vesicular GTPase Rab3 with the plasma membrane-associated protein Rim (tethering). Subsequent activation of Munc18, which triggers the de-chaperonning of syntaxin, leads to SNARE complex assembly (priming/docking). Ca^++^ entry is sensed by synpatotagmin and triggers the fusion event and release of neurotransmitter through Rac-PLD1 activation. Clostridial neurotoxin L chains have access to the cleavage sites of SNARE proteins when the SNARE proteins are dissociated (physiological window). Cleavage of SNAP25 by BoNT/A or BoNT/C, SNARE complex formation still occurs. But, the release of the C-terminal domain of SNAP25 impairs the interaction between SNAP25 and Ca^++^ to synaptotagmin thus preventing the fusion. Truncation of SNAP25 by BoNT/E or VAMP by BoNT/B, BoNT/D, BoNT/F or TeNT destabilizes or leads to non functional SNARE complexes. LT acts differently by glucosylating and thus inactivating Rac and subsequently preventing the PLD1 production of fusogenic phosphatidic acid.

L chains of all clostridial neurotoxins are zinc-metalloproteases that cleave one of the three members of the SNARE proteins. TeNT and BoNT/B, D, F and G attack synaptobrevin (or VAMP), BoNT/A and E cleaves SNAP25, and BoNT/C1 cut both SNAP25 and syntaxin. The cleavage sites are different for each neurotoxin except BoNT/B and TeNT, which proteolyse synaptobrevin at the same site. Cleavage of SNARE proteins occurs only when disassembled. Since VAMP, SNAP25, and syntaxin play a major role in the regulated fusion of synaptic vesicles with the plasma membrane at the release sites, their cleavage induces a blockade of the neurotransmitter exocytosis (Figure 2).

SNAP25 cleavage by BoNT/A or BoNT/E deeply decreases both SNAP25 and Ca^++^ binding to synaptotagmin, and subsequently the fusion process of exocytosis (Figure 2) [179,180,181,182]. Removal of the nine C-terminal amino acids of SNAP-25 by BoNT/A deeply disrupts the coupling between Ca^++^ sensing and the final step in exocytosis [180]. Truncated SNAP-25 can behave as a dominant negative mutant upon the exocytotic process suggesting that after BoNT/A treatment, the block of release is due to both functional elimination of SNAP-25 and accumulation of the cleavage product which competitively inhibits exocytosis [183,184,185]. In contrast, blockade of exocytosis by BoNT/E is only due to cleavage of SNAP-25, not to the production of competitive antagonists of SNARE complex formation. Indeed, inhibition of exocytosis by BoNT/E can be rescued by supplementing the C-terminal portion of SNAP-25 removed by the toxin [186,187,188]. Truncation of SNAP-25 by BoNT/E destabilizes the four-helix bundle of the SNARE complex [186,187], and SNAP-25 truncated by BoNT/E is not retained by syntaxin [189].

VAMP cleavage abolishes the interaction of VAMP with the adaptor protein AP3 and affect synaptic vesicle recycling *via* early endosomes [190]. The SNARE cleavage products have also the potential to interfere with fusion processes [179,191]. Consistent with synaptophysin-1 controlling specifically the targeting of VAMP2 but not VAMP1 to synaptic vesicles, is the observation that the cytosolic cleavage product of VAMP2 but not VAMP1, released upon TeNT or BoNT/B activity, blocks the neurotransmitter release [191]. This result suggests an alteration of the exocytosis due to a disturbance of synaptophysin-1/VAMP2 interaction and of coupling between detecting Ca^++^ and synaptic vesicle triggering [180]. Since the synaptic vesicles docked with unproductive complexes cannot fuse or undock, they stay at the fusion sites (with slightly increased numbers) irreversibly plugging the fusion sites that would normally accommodate intact vesicles. This progressively reduces the number of release sites to which exocytosis can occur as recently demonstrated for TeNT at identified *Aplysia* cholinergic synapses [36]. When VAMP is cleaved by TeNT, BoNT/B or /G, the VAMP portion (~20 amino acids) remaining in the synaptic vesicle membrane does not contain interaction sites for the other SNAREs. Therefore, the synaptic vesicle membrane is no longer linked to a SNARE complex, and fusion with the plasma membrane cannot occur. When VAMP is cleaved by BoNT/D or /F, the C-terminal fragment remaining in the vesicle membrane is long enough to anchor the synaptic vesicle to the SNARE complex, but fusion cannot occur because the SNARE complex cannot transit into the thermally stable four-helix bundle (Figure 2). 

BoNT/C cleaves both syntaxin-1 and SNAP-25, but *in vitro* cleavage of SNAP-25 by BoNT/C occurs with low efficiency (~1000-fold difference) versus cleavage by BoNT/A or /E [192,193]. This raises the question which of the two targets is involved in BoNT/C neuroexocytosis blockade? In squid giant synapses, BoNT/C cleaves syntaxin-1, but not SNAP25 [194], whereas in cultured hippocampal slices or spinal neurons from mammal, BoNT/C efficiently removes nearly all SNAP25 [195,196]. Thus, depending on the cell type, the secretory blockade is likely due to syntaxin and/or SNAP-25 cleavage. Upon syntaxin cleavage, SNARE complexes are formed, but loosely docked to plasma membrane, thus synaptic vesicles remain tethered to plasma membrane and cannot fuse [44]. 

Although the physiological properties induced by the cleavage of either VAMP, SNAP25 or syntaxin are not equivalent at the neuromuscular junctions, all the clostridial neurotoxins cause a blockade of the regulated neurotransmission, which varies in intensity and duration according to each neurotoxin type. TeNT and BoNT/B share the same molecular mechanism. They are translocated in different subset of neurons (excitatory neuron: BoNTs >> TeNT; inhibitory neurons: TeNT >> BoNTs) produce strongly different symptoms. This induces different clinical signs (TeNT: spastic paralysis; BoNTs: flaccid paralysis). Indeed the peripheral dysautonomia and flaccid paralysis caused by BoNTs result from preferential inhibition of acetylcholine release. In the spinal chord or facial motor nuclei, TeNT-mediated blockade of glycine or GABA release disrupts the negative controls exerted by the inhibitory interneurons onto the motoneurons turning on excessive firing of the motoneurons and ensuing muscle contraction [42,44,45,197].

#### 3.1.3. Duration of intoxication

The main factor governing the duration of intoxication is the BoNT toxinotype. The half-lives of exocytosis blockade in rat cerebellum neurons are more than 31 days for BoNT/A, more than 25 days for BoNT/C1, about 10 days for BoNT/B, about two days for BoNT/F and less than one day for BoNT/E, and these durations correlates with the paralysis duration [97,198,199]. The factors governing the toxin longevity within the nerve terminals are not fully unraveled, yet presence of a N-terminal sequence and a C-terminal dileucine motif in BoNT/A L chain, and not in the other toxinotypes, may explain the retention of BoNT/A L chain to the plasma membrane [200]. Other factors should be considered: in contrast to BoNT/E truncation, SNAP25 lacking nine C-terminal residues, released by BoNT/A proteolysis, remains localized at the membrane in association with syntaxin. Therefore, unproductive SNARE complex at the active zone induces long-term impeding of synaptic vesicle fusion, whereas SNAP25 cleaved by BoNT/E cannot bind to syntaxin and is released into the cytosol [189,200].

To summarize, despite the scenario that the blocking actions of the various BoNTs differ at the molecular level, they all share several commonalities. Indeed, in all cases following toxin action, the formation or stability of the SNARE complex is compromised [201,202], and it is no longer fusogenic. Thus, synaptic vesicles remain docked at the fusion sites with slightly increased numbers, suggesting that when tethered to the plasma membrane or docked in an unproductive complex, not only exocytosis but also undocking cannot proceed. Therefore, it is likely that synaptic vesicles docked with unproductive complexes can irreversibly plug the fusion sites that would normally accommodate intact vesicles.

#### 3.1.4. Other consequences of SNARE cleavage

SNAREs are able to interact, directly or indirectly, with other proteins present in the plasma membrane. Their cleavage would indirectly affect the functioning of their partners. For example, syntaxin promotes inactivation of N- or P/Q-, R-, L-types Ca^++^ channels, and this inactivation is removed when proteins of the fusion complex (SNAP-25, VAMP, synaptotagmin…) bind to syntaxin and/or Ca^++^-channels [203,204,205,206]. In this line, SNAP-25 has been reported to control calcium responsiveness to depolarization [122]. Syntaxin cleavage may indirectly alter Ca^++^-channel functioning: slow Ca^++^ influx is potentiated after BoNT/C [207], while in *Torpedo* preparations, Ca^++^ entry was reported to be significantly decreased by BoNT/C [208]. Moreover, BoNT/C abolishes the regulation of Ca^++^-channel by heterotrimeric G-proteins [209]. SNAP-25 modulates L-Type channels [210], and entry of Ca^++^ mediated by store operated-channels in pancreatic acini secreting amylase, or human platelets. In these cells, Ca^++^ influx is strongly depressed after SNAP-25 cleavage by BoNT, possibly contributing to the blockade of Ca^++^-dependent exocytosis by BoNT/A [130]. Note however that these non-neuronal effects have been detected using very high concentration of toxin (~100 nM). Overall, these observations suggest that in certain preparations, the blockade of secretion resulting from SNAP-25 cleavage can be related to inhibition of the exocytotic machinery and changes in Ca^++^ influx at the nerve terminals. A significant reduction of the store operated Ca^++^ entry (SOCE) due to BoNT/A has been observed in different cells. SOCE normally follows a depletion of intracellular Ca^++^ stores ant this Ca^++^ influx is carried out by store-operated Ca^++^ channels (SOCC) [108,211,212]. Working with *Xenopus* oocytes, Yao *et al.* demonstrated that the activation of a store-operated Ca^++^ current requires SNAP-25, since the current was found to be inhibited by BoNT/A in a dose-dependent manner with an apparent Ki of 8 nM. Similarly, the store-operated Ca^++^ current was almost completely abolished by expression of C-terminal truncated SNAP-25 mutants [213]. BoNT/A L chain and TeNT significantly reduce SOCE when directly microinjected in human embryonic kidney (HEK) cells [214]. SOCE is also significantly reduced by BoNT/A or BoNT/E in murine pancreatic acinar cells and in human platelets [130,131]. The molecular identity of the channel carrying the Ca^++^ current in SOCE is in the process of clarification, then, it remains difficult to assert a role to SNAP-25 in the regulation of the conductance or in the process of protein secretion. A direct interaction of SNAP-25 with TRPC1 channel has been postulated [215] and, more recently, the inhibition of exocytotic-like insertion of Orai1 into the plasma membrane in human HeLa and HEK 293T cells has been shown to be partially affected by BoNTA [216]. Likewise, physical and functional interactions between syntaxin 1A and brain voltage gated K^+^ -channels Kv1.1 type have been found [217]. The activity of Kv2.1 channel, the prevalent delayed rectifier channel in endocrine and neuroendocrine cells, is also strongly modulated by syntaxin and SNAP-25 [218,219]. This suggests that SNARE proteins can regulate membrane excitability *via* K^+^ channels, thus tuning exocytosis. This raises the question of whether exocytosis may be altered by BoNTs *via* changes in activation of Kv2.1 currents.

#### 3.1.5. Non-proteolytic molecular actions of BoNTs and TeNT

Yet a direct cause-effect relationship exists between the cleavage of the SNAREs and the blockade of neuroexocytosis by BoNTs or TeNT [41,44,220]. A controversial possibility is that BoNTs and TeNT may interfere with exocytosis and other cell functions *via* molecular actions unrelated to their proteolytic activity. Indeed, when mutated in the catalytic site at positions crucial for either Zn^++^ binding (His233 and His237) or cleavage of the Gln-Phe bond in VAMP-2 (Glu234), TeNT L chain cannot cleave VAMP-2 *in vitro* [221,222]. However, several of point-mutated TeNT L chain constructs are able to produce inhibition of neurotransmitter release (His233- > Ala233, Leu233 or Val233; Glu234- > Ala234; His237- > Ala237; Asp237, Gly237 or Val237) albeit with reduced potency as compared to wild-type TeNT L chain [223,224]. Such a non-proteolytic mechanism may explain why endopeptidase blockers, which abolish VAMP-2 cleavage *in vitro*, counteract only partially the inhibitory action of TeNT on neurotransmitter release [223,225]. Moreover, the observation that antagonism of the intracellular action of BoNT/A can be relieved fast by the mean of injecting monoclonal antibodies directed against the BoNT/A L chain [226] is difficult to conciliate with the proteloytic activity of the neurotoxin protein cleavage being in essence irreversible. The observation that TeNT binds with high affinity to, and strongly activates the GTP-binding protein transglutaminase type II (TGase II) *in vitro*, suggests that TGase II may participate in the intracellular action of TeNT [227,228]. However, the precise contribution of TGase II to the blockade of neurotransmission by TeNT has never been clarified and conflicting data exist in the literature [223,229,230]. TGase II belongs to a large family of bifunctional and Ca^++^-dependent cross-linking enzymes [231,232] abundant in neurons and nerve endings [228,233] which has been implicated in secretory mechanisms [234,235,236]. 

The identification of the vesicular protein synapsin-I as one of the two main substrates crosslinked by TGase II needs to be considered for explaining part of the nonproteolytic TeNT-induced decrease in neurotransmitter release. Indeed, synapsin-I regulates synaptic vesicle trafficking *via* interactions with the actin cytoskeleton and participates in post-docking steps of exocytosis [237,238]. Possibly, TeNT stimulation of TGase II leads to reduced synaptic vesicle availability for release. This view is supported by several observations: (i) the depolarization-stimulated phosphorylation and redistribution of synapsin-I is altered after the action of TeNT [239]; (ii)the blocking action of TeNT is diminished after disassembly of microfilaments [150] and (iii)the amplitude of post-tetanic potentiation, a plasticity paradigm which involves synapsin-I in *Aplysia* synapses, is highly reduced after TeNT treatment [36,237]. As TeNT can access VAMP-2 only during a defined “physiological window” [41,44], TGase-II activation may modulate this access *via* the modification of proteins involved in regulation of the synaptic vesicle cycle (*i.e.*, synapsin I) or its other substrate(s). 

The importance of the proteolytic and non-proteolytic mechanisms of TeNT may be variable from one model systems to other, and may depend on differential expression of endogenous TGase-II. Contrasting with the observations made using brain synaptosomes or *Aplysia* preparations [223,224], non-proteolytic TeNT mutants have been found ineffective at the mouse hemidiaphragm [221] or neurohypophysial nerve endings [240] and participation of TGase-II activation in the blockade of secretion by TeNT has been ruled out at the mouse neuromuscular junction and in NG108 cells [229]. *In vitro*, BoNT/E light chain has been reported cleaving actin and all the 11 cleavages sites identified involved Arg or Lys residues in *P*’1 position exactly as in SNAP-25 [241]. Thus another unexpected intracellular effect of TeNT is the modification of actin cytoskeleton. This is supported by several observations: TeNT inhibits the rearrangements of subcortical microfilaments that accompany secretion in chromaffin cells [242]. Actin cytoskeleton network is altered when TeNT L chain is expressed in Sertoli cells in mice [243]. Consistent with the well documented implication of small GTPases Rho in the dynamics and organization of actin-based cytoskeleton [24], BoNT/A has been reported to target RhoB to the proteasome, causing both blockade of exocytosis and actin cystoskeleton disorganization [244]. This may relate to a crosstalk between actin cytoskeleton remodeling, SNARE- and Rho-GTPase-dependent mechanisms of exocytosis, as illustrated for Cdc42 and VAMP-2 during insulin secretion [245]. 

TeNT shows unconventional cellular action time before its classical proteolytic effects became evident. Aguliera and Yavin first reported the *in vivo* activation and translocation of protein kinase C in rat brain [246]. Concomitantly, an increase of phosphoinositide hydrolysis was observed [247]. The intracellular pathway activates phospholipase C-1 and other kinases [248]. Among the different targets these enzymes can attain, the best characterized is the 5-HT transporter which is phosphorylated and has its activity modulated at low toxin concentration (10^–12^ M) in less than 30 minutes [155,157,158]. These effects are carried out by the half C-terminal part of the H chain, the portion that carries the binding domain of the toxin to the receptor [249,250]. It is of interest to note that this portion of the H chain is able to protect from death neuronal cells, *in vitro* as well as *in vivo* [250,251,252]. Indeed, TeNT Hc activates phosphatidylinositol 3-kinase (PI-3K)/Akt (a serine/threonine kinase) as well as extracellular-signal regulated kinases 1 and 2 (ERK-1/2) pathways through phosphorylation of tyrosine kinase receptor leading to protection of apoptosis by preventing the proteolytic activation of pro-caspase-3, cytochrome c release from mitochondria, and chromatin condensation [249,251,253]. Interestingly, TeNT Hc prevents apoptosis induced by 1-methyl-4-phenylpyridinium (MPP+), which is a mitochondrial poison used to reproduce a Parkinson-like disease [251]. Since TeNT Hc is retrogradely transported to the central nervous system it could be used in the prevention/treatment of the Parkinson's disease. *In vivo* in a rat model of Parkinson's disease, TeNT HC has been found to improve the dopaminergic system and to enhance the survival rate [252].

### 3.2. Toxins which inactivate Rho-GTPases and inhibit neuroexocytosis

#### 3.2.1. Glucosylating clostridial toxins

Glucosylating clostridial toxins, also known as the large clostridial toxins such as *Clostridium sordellii* lethal toxin (LT) and *Clostridium difficile* toxin A (ToxA) and toxin B (ToxB), inactivate Rho-and/or Ras-GTPases by modifying a conserved threonine within the effector domain of target molecules. ToxB catalyzes glucosylation of Rho proteins (Rho, Rac, and Cdc42), whereas the several variants of LT modify Rac, sometimes Cdc42, and various Ras proteins (*i.e.*, Ras, Rap, Ral). *C. difficile* and *C. sordellii* toxins use UDP-glucose as cosubstrate, and *Clostridium novyi* α-toxin uses UDP-*N*-acetylglucosamine. GDP-bound GTPase is the preferred substrate, since the modified Thr35 and Thr37 residues are only exposed on the surface of this GTPase form. 

Although glucosylated GTPases can bind to their target membrane and undergo GDP-GTP exchange when stimulated by guanylate exchange factors, modification of Thr37/35 which is localized within switch I of GTPases, prevents the interaction with their effectors. In addition, intrinsic GAP (GTPase activating protein)-stimulated GTPase activity is reduced, inhibiting the GDP-GTP cycling of modified GTPases between the membrane and cytosol. Thereby, glycosylated GTPases act as dominant negative mutants [254,255,256,257,258,259,260].

*C. botulinum* C3 enzyme is an ADP-ribosyltransferase, which specifically inactivates Rho. C3 ADP-ribosylates Asn41, which is localized within the switch I loop near Thr37. C3-mediated ADP-ribosylation of Rho inhibits the GTPase translocation to the membrane and therefore prevents the interaction with its effectors. ADP-ribosylated Rho is trapped by guanine dissociation inhibitor (GDI) in an inactive, cytosolic form [24,260,261,262].

#### 3.2.2. Neuronal alterations caused by toxin inactivation of Rho GTPases

Several small GTPases of the Ras superfamily are present in nerve terminals and include RhoA, RhoB, Rac1, and Cdc42. Additionally, Rac1 associates with synaptic vesicles as well as with plasma membrane [27,28]. The Ral molecule also binds to synaptic vesicles [31]. Intraneuronal application of C3 exoenzyme, ToxB from *C. difficile*, or LT from *C. sordellii* blocks Ca^++^-dependent vesicular ACh release from *Aplysia* synapses [27,28]. LT exhibits the highest activity upon neurotransmitter release, being 100–1,000 fold more potent than ToxB or the C3-exoenzyme [27]. Similarly, LT blocks spontaneous glutamate release in cultured rat cerebellar slices (Kojima and Poulain, unpublished observation). These observations are consistent with reports that Rho-acting toxins block the secretory process in many cell types, including mast cells [263,264,265,266]. In muscles isolated from mice previously injected with LT, nerve-evoked muscle twitch is blocked. This effect results from both a presynaptic block of ACh release and depressed muscle contractility [267]. 

This is consistent with the high lethality of LT (~0.3 × 10^6^ mouse LD50/mg protein) injected intraperitoneally, which is due to respiratory failure [268]. Because of the potent blocking action of LT on nerve endings, a question arises whether the intestinal disorders which characterize enterotoxemia caused by clostridia producing Rho-acting toxins are linked to intestinal epithelial damage and/or stimulation of the enteric nervous system [269]? Identity of the downstream pathways whose silencing blocks exocytosis induced by LT or ToxB has been addressed in different cell systems. Apparently, the affected pathways can vary in different cell types. In several types of secretory cells, experimental findings indicate that the toxins induce remodeling of the actin cytoskeleton, thereby altering intracellular granule trafficking towards the plasma membrane, which possibly includes fusion [265,266]. Another interesting configuration has been deciphered in RBL-mast cells. In these cells, exocytosis is triggered by a rise in intracellular Ca^++^ levels induced by stimulation of either the Fc-epsilon receptor or muscarinic- type AChR. The Rac GTPase is the only one involved in Ca^++^ mobilization within these cells, and the blockade of exocytosis by ToxB or LT is due to inhibition of capacitative Ca^++^ entry exclusively through store-operated Ca^++^ channels [263,270]. 

In nerve endings, the blockade of neurotransmitter exocytosis caused by ToxB and LT appears to have another origin. Studies performed with *Aplysia* cholinergic synapses reveal that following inactivation of Rac by LT or ToxB, the exocytosis process starts normally, but then stops, possibly during the priming events. This results in a dramatic decrease in the number of functional release sites [28], which is very similar to the gross action of TeNT or BoNTs. What is the role of Rac during priming? Rac, Rho, Cdc42, Ral, and PLD1 all participate in a multiprotein complex termed “exocyst,” which is comprised of Sec6/Sec8 proteins and regulates polarized secretion as well as docking of vesicles to plasma membrane regions specialized in exocytosis [271]. Therefore, one cannot exclude that Rho-acting toxins block exocytosis by disabling exocyst function in nerve endings. However, implication of an exocyst role during exocytosis of neurotransmitter is disputed. Alike, possibility implicating PLD1 in the exocytosis process is that by catalyzing PA generation, the formation of lipid bilayer intermediates becomes favored during membrane fusion. PLD1 activity is inhibited in LT-poisoned cells [272], and Rac plus Cdc42 regulate PLD1 activity [273]. The intraneuronal application of a catalytically- inactive mutant of PLD1 reproduces the potent inhibitory effect of LT [237]. Therefore, we envisage the following model for LT and ToxB action at the presynapse, which initially involves glucosylation of Rac by the toxins, thus preventing activation of PLD1 upon synaptic vesicle docking. Production of fusogenic PA is not stimulated, and despite the Ca^++^ influx triggering SNARE complex zippering, fusion of synaptic vesicle with plasmalemma exocytosis cannot occur because the lipid composition at release sites remains inadequate. The synaptic vesicles equipped with modified Rac plug exocytosis sites and prevent fusion of synaptic vesicles equipped with intact Rac (Figure 2). This contributes to the reduced numbers of active release sites induced by LT or ToxB. In addition to the presynaptic actions mentioned above, clostridial cytotoxins affecting Rho GTPases can also block neurotransmission by altering postsynaptic cell capabilities to detect transmitter. For example, neurotransmitter-receptor density is diminished in neurons treated with toxins that affect Rac or Cdc42 [274]. The observation that LT prevents long-term potentiation [275] is consistent with inhibiting insertion of new receptors into the post-synaptic membrane. Note that it is not yet clear whether changes in receptor density refer to the role of Rho proteins during the exocytotic process, which includes receptor insertion into the plasma membrane, and/or organization of the post-synaptic actin cytoskeleton involved in receptor clustering [276]. 

Much evidence suggests that apoptosis plays a crucial role in cell homeostasis, which depends upon the expression of various genes implicated in controlling life and death. Incubation of rat primary cerebellar granule neurons in culture with either ToxB or LT reportedly induces cell death with biochemical and morphological hallmarks of neuronal apoptosis [277]. In these cells, selective inhibition of Rac/Cdc42 function promotes: (1) phosphorylation and expression of the transcription factor c-Jun; (2) activation of caspase-3; and (3) nuclear condensation, as well as fragmentation. Interestingly, apoptosis occurs independently of F-actin-cytoskeletal disruption, since agents that directly disassemble F-actin (*C. botulinum* C2 toxin, cytochalasin D, and latrunculin A) do not induce cell death within 24 h. These results indicate that Rac/Cdc42 GTPases are critical for survival of cerebellar granule neurons in primary culture. Additionally, these data are the first to establish a prosurvival function for Rho GTPases in a primary neuronal cell model.

### 3.3. Toxins which damage neuronal cells, neuronal apoptosis

*Strpetococcus pneumoniae* (or *Pneumococcus*) is the most common cause of bacterial meningitis, which is characterized by neuronal cell death in the hippocampus as well as by an inflammatory response. Pneumolysin (PLY) produced by *S. pneumoniae*, is one of the virulence factors leading to neuronal cell damages and which also causes injury to pulmonary alveolar epithelial cells [278].

PLY (53 kDa) is a pore-forming toxin from the cholesterol-dependent cytolysin family, the prototype of which is the perfringolysin (PFO) from *C.* *perfringens*. PLY shares 45% identity at the amino acid level with PFO. In contrast to PFO, PLY contains no signal sequence for secretion and is released mainly during autolysis of bacteria [279]. A structural model of PLY based on the high similarity with PFO shows that PLY has an unusual elongated rod shape. The molecule is rich in β-sheet and it is hydrophilic without significant patches of hydrophobic residues on the surface. Four domains can be distinguished in the PFO or PLY molecule. Domain 1 has a seven-stranded antiparallel β-sheet and is connected to domain 4 by the elongated domain 2. Domain 3 consists of β-sheets and α-helices. The C-terminal part (domain 4) folds into a separate and compact β-sandwich domain, and contains three loops (L1-L3), which are involved in the binding to choleterol [280,281]. 

A unique Cys is located in a conserved 11 amino acid sequence (ECTGLAWEWWR) near the C-terminus, in the domain 4, which participates to the prepore to pore conversion [280,282]. The proposed model of PFO pore formation, which is probably similar to that of PLY, includes the binding of water soluble PFO monomers to cholesterol of lipid bilayer mediated by the L1-L3 loops from domain 4 [280]. But, domain 4 does not insert deeply into the membrane and is not directly involved in creating the pore. PFO monomers bound to cholesterol and orientated perpendicularly to the membrane assemble and oligomerize to form a prepore complex [283]. Oligomers consist of 40 to 50 monomers forming on the membrane surface large arcs and rings leading to large pores between 300 Å and 450 Å in diameter [284]. Domains 1, 2 and 4 fit into L-shaped repeating units connected to the corresponding domains of the neighboring partners and forming a cylindrical structure. Oligomer formation results from domain 1-domain-1 interaction *via* hydrogen bonding between 1-strand of one subunit with 4-strand of a second subunit. Interaction of domain 4 with cholesterol induces a conformational change of domain 1 causing the moving of β5-strand which prevents 1-4 interaction in the soluble PFO form and thus permitting the oligomerization process only when PFO interact with cell membrane [285,286]. Thereby, PFO monomers do not oligomerize in solution. In addition, domains 3 are rotated from domains 2 and form a belt in the outside face of the cylinder. This is accompanied by a flexing of domain 2 leading to a loss of many contacts between domain 3 and domain 2 thus promoting the exposure of hydrohobic residues and the insertion of a transmembrane β-barrel into the lipid bilayer [287,288,289]. A bundle of three α-helices of domain 3 unfolds forming two amphipathic β-sheets. Each monomer contributes two amphipathic β-hairpins to the formation of the transmembrane β-barrel [290,291]. Monomers do not insert their transmembrane hairpins individually, but a cooperation between PFO monomers is required to drive the insertion of the prepore complex, which appears to be an all or none process [292]. The charged face of domain 4 amphipathic β-hairpin forms the inner lining of the pore and the other face is protected from the hydrophobic part of the lipid bilayer by cholesterol molecules [293]. Domain 2 collapses vertically of 40 Å allowing the insertion of the β-barrel into the membrane and formation of a large membrane pore [294].

An initial step in bacterial meningitis is the passage of *S. pneumoniae* from blood to the brain through the blood brain barrier. PLY is probably involved in this process, since PLY expressing strain but not PLY negative mutant is able to penetrate into microvascular endothelial cells. The mechanism of PLY-dependent translocation of bacteria across the cell barrier is possibly based on loss of cell viability and cell detachment [278,295]. Subsequently in the central nervous system, PLY directly induces hippocampal neuronal cell death *via* an apoptotic process. Indeed, PLY was found to colocalize with damaged neurons in the hippocampus and to mediate microglia and neuronal cell apoptosis *in vitro* [296]. The PLY pore-forming activity is required to trigger apoptosis in primary hippocampal neurons, whereas the PLY domain involved in complement activation is not required. The PLY-dependent pathway leading to neuronal cell apoptosis includes an increase in Ca^++^ into the cytosol subsequently to an influx from extracellular space and a release from endoplasmic-reticulum stores, mitochondrial damages with release of cytochrome *c* and AIF (apoptosis inducing factor) and then DNA fragmentation [296,297]. The increase in intracellular Ca^++^ seems to be mainly mediated by toxin-dependent pore formation in cell membranes and is followed by an activation of p38 MAPK (mitogen-activated protein kinase) and possibly by opening of the mitochondrial permeability transition pore leading to AIF release [298]. However, PLY also directly interacts with mitochondria as evidenced by colocalization of PLY with mitochondrial membranes, and can possibly directly forms pores in mitochondria. PLY apoptosis is caspase-independent and is mediated by the release of mitochondrial AIF, which is a potent caspase-independent cell death factor [296,297]. Thus PLY is a pore-forming toxin inducing apoptosis in hippocampal neuronal cells in addition to damages caused in other cell types.

## 4. Toxins Which Stimulate Neurosecretion

### 4.1. Stimulation of glutamate release: Clostridium perfringens epsilon toxin

Epsilon toxin acts on different cell types, including those of neuronal origin. It is not a strict neurotoxin as defined previously for BoNTs and TeNT, but epsilon toxin is involved in neurological symptoms. Epsilon toxin represents the major virulence factor of *C. perfringens* types B and D, which are the etiological agents of fatal enterotoxemia in sheep as well as goats, and more rarely of cattle. Overgrowth of *C. perfringens* in the intestine of susceptible animals, generally a consequence of overeating high starch/sugar-containing foods, results in large amounts of epsilon toxin. The toxin is absorbed through the intestinal mucosa by opening tight junctions between intestinal epithelial cells and spreads into different organs by the circulatory system [299,300], ultimately causing hypertension, increased vascular permeability, lung edema, and post mortem kidney necrosis (pulpy kidney disease in lambs) [260,301,302]. The disease is rapidly fatal with neurological disorders that include excitation symptoms. Major pathological changes are observed in the brain following epsilon intoxication, which include: congestion and edema of the meninges, perivascular and intercellular edema, as well as necrotic foci of the nervous tissue. Epsilon toxin passes through the blood brain barrier and accumulates specifically in the brain [303,304]. The toxin damages endothelial cells of the blood-brain barrier by altering the endothelial barrier antigen localized to luminal endothelial cell membranes, thus causing leakage of endogenous albumin [305]. The specific receptor for epsilon toxin on neuronal and endothelial cells is not yet known, although a membrane sialoglycoprotein seemingly mediates toxin binding [304,306]. In the brain, epsilon toxin increases vascular permeability leading to a rapid, severe, and diffuse edema that exerts a direct cytotoxic effect upon neurons. Neuronal damage is characterized by progressive cytoplasmic vacuolization plus necrosis, and in some cells there is hyperchromatosis and nuclear pyknosis [307]. The neurological disorders linked to epsilon toxin, which likely result from an excessive release of glutamate, include retraction of the head, opisthotonus, convulsions, agonal struggling, and hazardous roaming. Indeed, epsilon toxin injected intravenously into mice preferentially excitates hippocampal neurons that induce an increased glutamate efflux which can be blocked by a specific inhibitor [308,309]. 

The molecular basis for epsilon neurotoxicity is not known, but the toxin is a pore former, which structurally mimics *Aeromonas hydrophila* aerolysin [310]. Epsilon-toxin retains an elongated form and contains three domains, which are mainly composed of β-sheets. The overall structure is significantly related to that of the pore forming toxin aerolysin. The main difference between both toxins is that the aerolysin domain I, which is involved in initial toxin interaction with cells, is missing in epsilon-toxin. Domain 1 of epsilon-toxin consists in a large α-helix followed by a loop and three short α-helices and is similar to domain 2 of aerolysin which interacts with the GPI anchors of proteins. This domain of epsilon-toxin could have a similar function of binding to receptor. Domain 2 is a β-sandwich structurally related to domain 3 of aerolysin. This domain contains a two-stranded sheet with an amphipatic sequence which is the channel-forming domain [311]. Domain 3 is also a β-sandwich analogous to domain 4 of aerolysin and contains the cleavage site for toxin activation. Domain 3 after removing of the C-terminus is likely involved in monomer-monomer interaction required for oligomerization [312].

In Madine Darby Canine Kidney (MDCK) cells, which is a rare cell line sensitive to epsilon toxin, the toxin recognizes a specific unknown receptor, heptamerizes, and forms pores that lead to an efflux of K^+^, influx of Na^+^, Cl^−^ and Ca^++^, cell swelling, and membrane blebbing/disruption [313,314,315]. In addition, epsilon toxin rapidly decreases membrane barrier permeability of polarized MDCK cells [316]. Epsilon toxin also forms heptamers in synaptosomal membranes [313,317], which involves detergent resistant membrane microdomains called lipid rafts. But it is not yet defined whether the toxin-induced glutamate release is due to pore formation or another specific mechanism.

The epsilon toxin-dependent cytotoxicity is associated with a rapid loss of intracellular K^+^, and an increase of Cl^−^ and Na^+^, whereas the increase of Ca^++^ occurs later. In addition, the loss of viability also correlates with the entry of propidium iodide, indicating that the epsilon-toxin forms large pores in cell membrane. Pore formation was evident in artificial lipid bilayer. Epsilon-toxin induces water-filled channels permeable to hydrophilic solutes up to a molecular mass of 1 kDa, which represent general diffusion pores slightly selective for anions [315]. In polarized MDCK cells, epsilon-toxin induces a rapid and dramatic increase in permeability. Pore formation in the cell membrane is likely responsible for the permeability change of cell monolayers. Actin cytoskeleton and organization of tight and adherens junctions are not altered, and the paracellular permeability to macromolecules is not significantly increased upon epsilon-toxin treatment [318,319]. Epsilon toxin causes a rapid cell death by necrosis characterized by a marked reduction in nucleus size without DNA fragmentation. Toxin-dependent cell signaling leading to cell necrosis is not yet fully understood and includes ATP depletion, AMP-activated protein kinase stimulation, mitochondrial membrane permeabilization, and mitochondrial-nuclear translocation of AIF [319]. Therefore, epsilon-toxin is a very potent toxin, which alters the permeability of cell monolayers such as epithelium and endothelium causing edema and cell death and which induces glutamate release from hippocampal neuronal cells.

### 4.2. Stimulation of serotonin release and other neuromediators from the enteric nervous system

Increasing evidence suggests that some enterotoxins mediate diarrhea by not only acting directly upon enterocytes, but also by interfering/stimulating the enteric nervous system [269]. The enteric nervous system is comprised of the myenteric plexus which is primarily involved in motor control of the gut and of the submucosal plexus, which plays a central role in sensing the chemical status of the intestine and in regulating secretion. Neuronal cell bodies of the enteric nervous system (intrinsic afferent neurons, interneurons, motor- and secretory-neurons) are clustered in ganglia and connected to parasympathetic and sympathetic systems. More than 20 neurotransmitters have been identified in the enteric nervous system. Among them, substance P, ACh, GABA, glutamate, and 5-HT modulate motility, absorption, and secretion of the intestine [320,321].

#### 4.2.1. Bacterial enterotoxins inducing increased intestinal secretion

##### 4.2.1.1. Cholera toxin

Cholera toxin (CT) is produced by *Vibrio cholerae* and is responsible for cholera, which is a serious epidemic disease characterized by severe diarrhea and dehydration. CT consists of an A or enzymatic subunit (28 kDa), and 5 B or binding subunits (11 kDa each) assembled in a pentamer (AB5 structure). The A subunit is proteolytically activated by a *V.* *cholerae* endopeptidase into two components A1 (approximately 22 kDa) and A2 (approximately 5.5 kDa) which remain linked by a disulfide bridge. The carboxy-terminal part of A2 extends through the central pore of the B pentamer and is linked non-covalently to the B subunits. Heat labile enterotoxin (HLT) from *Escherichia coli* is highly similar to CT, 88% amino acid sequence homology and closely related crystalline structure [322]. However, LT induces a much less severe type of diarrhea.

Both toxins, CT and HLT, recognize the glycosphingolipid GM1 on enterocyte membrane. CT entry into cell and intracellularr trafficking has been extensively studied. The whole toxin bound to GM1 receptors is then internalized into endocytic vesicles [323]. GM1 directs the toxin into lipid-rafts from where it enters the Golgi *via* early and late endosomes [324]. CT enters cells *via* multiple ways including clathrin-dependent and –independent pathways. But transport of CT from plasma membrane to Golgi is mainly mediated by endosomes containing caveolin-1 and is independent of clathrin coated-pits [325]. In the perinuclear region of the Golgi, the A subunit dissociates from the B subunits and enters the endoplasmic reticulum *via* coatomer I-coated vesicles and uses the cell secretion system Sec60/61. The carboxy-terminal sequence of the A2 fragment contains an endoplasmic reticulum (ER) retention sequence (KDEL), which may rather function to efficiently retain the toxins in the ER, allowing the A1 chain to be transported into the cytosol [326,327].

The A1 fragment is responsible for the enzymatic activities of the toxin, including NAD hydrolysis in ADP-ribose and nicotinamide, and covalent transfer of ADP-ribose to Arg-187 of the subunit of stimulatory protein (Gs leading to stimulation of adenylate cyclase and elevated intracellular cAMP [324]. The increased cAMP levels induces an activation of protein kinase A, which subsequently phosphorylates numerous substrates in the cell including the major chloride channel called the cystic fibrosis transmembrane conductance channel (CFTR). This results in an active Cl^−^ secretion and a decrease of NaCl-coupled absorption by enterocytes [322,324].

But in addition, CT stimulates 5-HT release from enterochromaffin cells primarily localized at the base of the epithelial crypts of the intestine, probably *via* its effect on adenylate cyclase activation. Increased intracellular cAMP activates Ca^++^ influx through L-type calcium channel resulting in exocytosis of 5-HT containing granules [328]. Indeed, an increased 5-HT release into the intestinal tissue and lumen was evidenced in response to CT in human and animal models, as well as an inhibition of CT-dependent intestinal secretion by pharmacological inhibitors of 5-HT receptors. A direct binding of CT to enterochromaffin cells seems to trigger the degranulation and 5-HT release [329]. Thus, 5-HT_2_, 5-HT_3_ and 5-HT_4_ receptors have been found to be involved in CT-mediated intestinal secretion [329,330,331,332,333,334]. 5-HT receptors are classified in seven families and are distributed on enterocytes, myocytes and neurons of the gastrointestinal tract. Except the 5-HT_3_ receptor, which is a ligand-gate ion channel, the other 5-HT receptors are seven-transmembrane domain G-protein-coupled receptors. 5-HT_2_ receptors are located to myenteric and submucosal neurons, enterocytes, and longitudinal and circular muscle cells of the gastrointestinal tract. They participate to intestinal motility and to intestinal secretion *via* an arachidonic pathway. 5-HT_3_ receptors are expressed in neurons of myenteric and submucosal plexus, interstitial cells of Cajal, and muscle fibers of circular and longitudinal muscles, as well as in submucosa and mucosa. Activation of 5-HT_3_ receptors results in gastrointestinal contractions and intestinal chloride secretion, and in 5-HT release from enterochromaffin cells. 5-HT_4_ receptors have been identified in submucosal and myenteric plexus as well as in nerve fibers of intestinal circular muscles. 5-HT_4_ receptors have an important role in gastrointestinal functions such as activation of gastrointestinal motricity *via* enhancement of ACh and CGRP release as well as stimulation of chloride and bicarbonate secretion by enterocytes [335]. Therefore, CT-induced intestinal secretion is mediated by 5-HT release from enterochromaffin cells and in part from enteric neurons by the toxin (Figure 1). Released 5-HT induces water and electrolyte secretion *via* stimulation of 5-HT_2_ receptors on enterocytes and *via* activation of secretory reflex(es) through the enteric nervous system. 5-HT activates afferent sensory nerves mainly through stimulation of 5-HT_3_ and 5-HT_4_ receptors. These afferent neurons, probably from the submucosal plexus, contain ACh/substance P, ACh/CGRP, and dynorphin or glutamate as neurotransmitters. They project to the myenteric plexus and are also directly connected to secretomotor neurons [336,337,338]. Indeed, CT-induced secretion can be blocked by the ACh nicotinic receptor antagonist hexamethonium, by substance P antagonists as well as by inhibitors of substance P receptors such as neurokinin-1 and neurokinin-2 [5,338]. 

**Figure 3:**
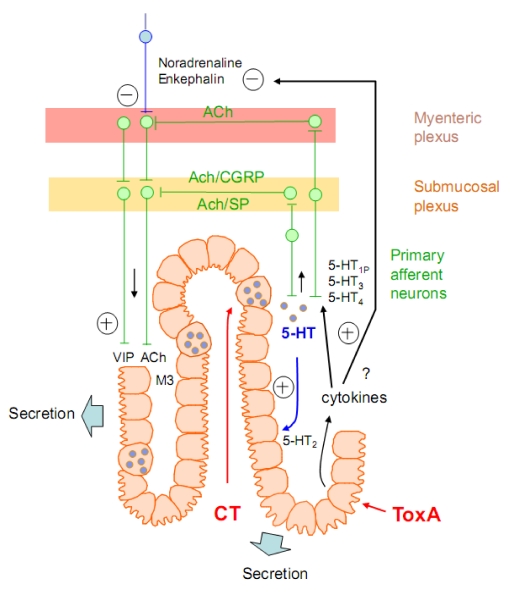
.Schematic model of the neuronal reflex controlling the intestinal secretion and alteration by enterotoxins. Cholera toxin (CT) triggers the release of serotonin (5-HT) from enterochromaffin cells, mainly located in intestinal crypts, which stimulates enterocyte secretion through 5-HT_2_ receptors and activates afferent sensory neurons through 5-HT_3_, 5-HT_4_ receptors. The neuronal signal is relayed through the submucosal and myenteric plexuses leading to activation of secretomotor neurons including acetylcholine (ACh) and vaso-intestinal peptide (VIP) neurons. ACh stimulates M2 receptors on enterocytes and VIP recognizes specific enterocyte receptors. The neuronal-dependent intestinal secretion is negatively regulated by noradrenergic and enkephalinergic neurons. *C.* *difficile* ToxA stimulates primary sensory neurons leading to the release of substance P (SP) and calcitonin gene-related protein (CGRP) and subsequently causing intestinal secretion and inflammation. ToxA first interacts with intestinal epithelial cells causing actin cytoskeleton alteration and release of cytokines, which are probably the stimulators of primary sensory neurons. In addition, ToxA attenuates the inhibitory response of noradrenergic and enkephalinergic neurons. The precise ToxA pathway permitting its interaction with the enteric nervous system is not yet known.

Cholinergic interneurons and possibly other interneurons containing other neurotransmitters probably connect the myenteric and the submucosal neurons of the reflex, which subsequently stimulate efferent secretory neurons releasing VIP and possibly nitric oxide (NO). VIP is a potent intestinal secretagogue, which recognizes specific receptors belonging to the type B G-protein coupled receptor family, on the basolateral membrane of crypt cells as well as of many other cell types, and induces an adenyl cyclase-cAMP-dependent secretion of NaCl and water. VIP triggers increase in camp level only in crypt cells and not in villus cells. camp activates protein kinase A and subsequently a phosphorylation cascade leading to stimulation of the apical CFTR channel and chloride secretion [5,336]. Antagonists of 5-HT, substance P and VIP markedly reduce CT-induced secretion [320,337,338]. In addition, ACh is a calcium-dependent chloride secretagogue through activation of muscarinic receptors such as M3 or possibly through a direct delivery into epithelial cells and seems to be also involved in the CT induced intestinal secretion [5]. Therefore, CT probably stimulates the two types of efferent neurons of the intestinal secretory reflex pathway, cholinergic and VIP neurons contributing to the toxin induced diarrhea (Figure 3).

Interestingly, *E. coli* HLT does not stimulate the release of 5-HT from enterochromaffin cells. Thereby, HLT-induced secretion is not inhibited by inhibitors of 5-HT or substance P. However, lignocaine and the ganglionic blocker hexamethonium have a preventive effect, suggesting that the entero nervous system is also involved in the enteric activity of HLT, but *via* a distinct pathway than that mediated by 5-HT. Although highly related to CT, HLT exhibits significant structural differences and recognizes a low affinity glycoprotein receptor. This might account for the non activation of enterochromaffin cells by HLT and the 5-HT-mediated intestinal secretion induced by CT explains, at least partially, the severity of cholera versus the more moderate diarrhea caused by HLT [320,329].

##### 4.2.1.2. Enterotoxins modifying the actin cytoskeleton or inducing an intracellular second messenger

*C. difficile* toxin A (ToxA; ~300 kDa) is a potent enterotoxin responsible for pseudomembranous colitis. In addition to the apparent actin-cytoskeleton alterations in enterocytes like ToxB, ToxA probably uses a neural mechanism to increase intestinal secretion (Figure 3). Lignocaine, hexamethonium, and substance P inhibitors can block Tox A-induced ileal fluid accumulation, mucosal permeability, and inflammation [339]. It is proposed that ToxA stimulates primary sensory neurons in the intestinal mucosa, causing subsequent release of substance P and CGRP, which then facilitates intestinal secretion and inflammation. These neuropeptides bind to macrophages in the lamina propria and activate the release of proinflammatory cytokines such as tumor necrosis factor (TNF) [269,340]. Thereby, ToxA stimulates intestinal macrophage expression of neurokinin-1, which is a high affinity receptor for substance P, and additionally neurokinin-1 deficient mice are protected from ToxA-induced intestinal secretion and inflammatory diarrhea [341]. Neurotensin, a gastrointestinal neuropeptide, is also involved since an inhibitor of neurotensin prevents the secretory and inflammatory responses following ToxA exposure [342]. Furthermore, ToxA also suppresses noradrenaline release at sympathetic synapses of the enteric nervous system that play an inhibitory effect on secretomotor neurons, thus amplifying mucosal secretion [343]. It is not yet clear whether ToxA directly stimulates intestinal sensory neurons by a mechanism involving Rho proteins, or indirectly *via* inflammatory mediators released from enterocytes or other cells such as macrophages in response to ToxA.

*C.* *perfringens* enterotoxin (CPE) is responsible for the food borne intoxication due to this bacterium. CPE is synthesized during the sporulation of *C.* *perfringens* and is a pore-forming toxin which binds to specific receptors on enterocyte membranes which have been identified to claudins leading to loss of fluid and electrolytes [344]. In addition, CPE interacts with nerve endings. Thereby, CPE has been found to bind to presynaptic nerve endings in mouse phrenic nerve-diaphragm, possibly at synaptophysin sites and to inhibit neurotransmission. CPE seems to block the neuroexocytosis process, by a yet unidentified mechanism [345]. The role of CPE interaction with the enteric nervous system in the diarrhea development is still unknown.

The much smaller *E. coli* heat stable enterotoxins (STa; 2–5 kDa) activate guanylate cyclase, increase cyclic guanosine monophosphate (cGMP) levels, and subsequently open Cl^−^ channels. Since STa-induced secretion is blocked by tetrodotoxin, lignocaine, hexamethonium, and also by capsaicin and vagotomy, the entero nervous system also likely supports STa activity [320,346]. The exact mechanism of activation of the enteric nervous system by STa is still elusive. VIP antagonist partially prevents STa-induced secretion indicating that VIPergic neurons are involved in STa enteric activity [347]. However in contrast to cholera toxin, STa seems not to stimulate 5-HT release [330,348]. But, STa would activate a NO-dependent myenteric plexus secretory reflex mediated by capsain sensitive C fibers [346,348]. VIP and NO seem to have a synergetic effect since NO releases VIP from nerve terminals and VIP seems to induce the release of nitric oxide, and both VIP and NO could participate in STa enteric activity [330]. STa might also activate neurokinin receptor NK2 to induce intestinal secretion [349].

#### 4.2.2. Emetic toxins

##### 4.2.2.1. *Bacillus cereus* cereulide

*Bacillus cereus* is a common foodborne pathogen, which causes two types of gastrointestinal diseases in humans, the diarrhoeal and the emetic syndromes. The diarrhoeal disease is due to enterotoxins, which are multicomponent toxins produced *in situ* subsequently to an over proliferation of *B. cereus* in the small intestine, whereas the emesis syndrome results from ingestion of a *B. cereus* emetic toxin, named cereulide, which accumulates in contaminated foods [350]. 

Cereulide is a cyclic dodecadepsipeptide (1.2 kDa) which is synthesized by a nonribosomal peptide synthetase [351,352,353]. Cereulide is produced by only certain *B. cereus* strains which are associated to the emetic syndrome [354]. The toxin is stable to acid conditions, proteolysis and heat, and thus it is not degraded by gastric acid and digestive proteases. Cereulide is cytotoxic, it induces mitochondria swelling (reported first as vacuole formation) in Hep2 cells and necrotic cell death in porcine pancreatic Langherans cells, as well as causes emesis in experimental animal models [355,356,357,358]. Cytotoxicity towards various primary or cultured cell lines has been reported [359]. The mechanism of action of cereulide is only partially known. Cereulide is a K^+^ ionophore similarly to valinomycin, as evidenced by selective increase in K^+^ permeability of phospholipids bilayers induced by the toxin [360]. Both toxins are also structurally related [352]. While valinomycin is more potent at high K^+^ concentration (120 mM), cereulide is more active than valinomycin at low K^+^ concentrations (1–3 mM) which corresponds to the physiological level in blood serum. Cereulide promotes K^+^ uptake by mitochondria, efflux of H^+^, drop in the transmembrane inner potential, leading to mitochondria swelling, arrest of respiratory function, inhibition of ATP synthesis, and release of proapoptotic or necrotic factors [360,361,362]. The emetic effects of cereulide seems to be dependent of stimulation of 5-HT_3_ receptors on vagal afferent neurons since 5-HT_3_ receptor antagonist such as ondanserom hydrochloride or vagotony inhibits the emetic effect in suncus [352]. It is not known whether cereulide directly interacts with vagal sensory endings or releases 5-HT from enterochromaffin cells.

##### 4.2.2.2. *Staphylococcus* enterotoxins

*Staphylococcus aureus* produces a myriad of 25–30 kDa, single-chain proteins called the *staphylococcal enterotoxins* (SEs) [363]. These potent toxins cause a prevalent form of food poisoning found throughout the world, and these molecules also possess “superantigenic” properties. By classic definition, this latter property involves binding to both major histocompatibility complex class II (MHC II) on antigen presenting cells and V-specific T-cell receptors, which ultimately cause massive T-cell proliferation with a concomitant uncontrolled production/release of various proinflammatory cytokines. To date, it is uncertain if superantigenicity plays a direct role in SE-induced food poisoning. However, levels of inflammatory mediators like prostaglandins and leukotrienes are increasingly evident in the circulatory system of non-human primates shortly after an oral dose of SE type B (SEB) [364]. Mast cells may also play a role in SE-induced food poisoning, which perhaps involves not only inflammatory mediators, but also stimulation by neuropeptides like substance P released from sensory neurons [365]. Another study reveals that SEB-induced effects in mice (intraperitoneal injection) are abrogated by capsaicin, the active ingredient of hot chili peppers, which depletes peptidergic sensory nerve fibers and TNF production [366]. An intraperitoneal injection of SEB into rats induces expression of Fos (a cell activator) throughout the brain *via* vagus nerve stimulation, thus suggesting that the peripheral presence of an SE has profound effects upon the brain [367]. More recently, it has been reported that SE type A (SEA)–induced emesis is mediated by 5-HT. Indeed, emesis caused by SEA in animal model (house musk shrew) is inhibited by 5-HT synthesis inhibitor and a 5-HT_3_ receptor antagonist, and in addition SEA increases 5-HT release in the intestine. The mechanism of 5-HT release mediated by SEA is not yet known. SEA might interact directly with enterochromaffin cells or neurons to trigger 5-HT release, or could act indirectly through the release of proinflammatory molecules or free-radical formation [368]. Mucosal terminals of vagal sensory afferent neurons, which project to the emetic center in the brainstem contains 5-HT_3_ receptors [335]. The proposed model includes a SEA-dependent excess release of 5-HT in the intestine and subsequent stimulation of 5-HT_3_ receptors on vagal afferent neurons triggering the emesis (Figure 1) [368].

## 5. Conclusions

Is neurotoxicity a unique, inherent function of a bacterial toxin? Most bacterial proteins with demonstrable “toxic” activity, such as those presented in this review, interact with various cell types. For example, more than one third of all bacterial toxins are pore-formers that recognize ubiquitous membrane components as receptors, such as cholesterol, gangliosides, and proteins. These toxins can indiscriminately damage membranes from different cells, including those of neuronal origin. Among them, *C. perfringens* epsilon toxin has the fundamental structure of a pore-forming toxin, is cytotoxic for kidney epithelial cells, and also possesses specific neurotoxic activity. However, a specific neurotoxic mechanism of action and trafficking pattern targeting neuronal cells by epsilon toxin during the natural course of disease still remains undetermined. Other toxins have developed various internalization processes, therefore specifically modifying an intracellular target. Thus, several bacterial proteins like the large clostridial toxins (*C. sordellii* LT and *C. difficile* ToxA/ToxB) have the ability to bind/enter various cell types that include those of neuronal origin. Large clostridial toxins inactivate intracellular targets like Rho and/or Ras GTPases, which are involved in multiple signaling pathways. Among them, Rac controls the neuroexocytosis process. Thereby, large clostridial toxins efficiently block neurotransmitter release, although they do not specifically target neuronal cells. Involvement of their neurotoxic activity during disease, which is often overcome by toxic effects upon other cell types in natural pathology, is also a function of a toxin’s opportunity to enter neuronal cells near the infection site. Various bacterial enterotoxins, which have developed a specific mode of interaction with enterocytes, amplify their intestinal activity by stimulating the secretomotor reflex from the enteric nervous system, like CT, or interact with distinct nervous afferences on the intestinal mucosa leading to vomiting. It is noteworthy that the enteric nervous system is a preferential target for many bacterial toxins, which transit through the intestinal tract. Finally, two unique classes of neurotoxins, BoNTs and TeNT, have evolved as specific inhibitors of the neuroexocytosis machinery. These bacterial proteins recognize specific receptors on neuronal cells and only interfere intracellularly with highly specialized molecules, like the SNARE proteins, which play a pivotal role in evoked release of neurotransmitter. It is intriguing that toxins produced by environmental bacteria, which are not normally adapted for a commensal life with higher organisms and only interact accidentally with them, possess such specific and highly sophisticated tools. What is the underlying selective pressure for such evolution from a bacterial protease to a neurotoxin? Perhaps neurotoxins simply result from an evolutionary process independent of the host. What is the inherited benefit derived by an environmental bacterium to produce a neurotoxin, which apparently does not recognize other bacterial or environmental substrates? Clearly, we approach this from a scientist’s (human) perspective, and with such an inherent bias that it may be difficult and virtually impossible to truly understand this natural curiosity. During their evolution, clostridial pathogens have developed a potent arsenal of noxious proteins that affect the central and peripheral nervous system of various vertebrates. These commonly named neurotoxins specifically target some key functions, or cellular processes, of eukaryotic cells, which subsequently cause a wide array of life-threatening diseases in humans and animals. These neurotoxins bind to specific receptors in the plasma membrane of susceptible host cells and translocate their enzymatically active domains/subunits into the cytosol, where finally they elicit the deleterious effects commonly associated with the holotoxins. Modes of action for the neurotoxic proteins reviewed here have been deciphered during the last decade, and now a clearer picture of their intracellular substrates is emerging. The specificity of these toxic proteins has enabled them to become useful tools to elucidate and characterize crucial processes for eukaryotic cells, which include neurotransmitter release, physiological signaling pathways, and constitutive cellular mechanisms. However, additional studies are needed to completely identify their specific membrane-receptors, as well as comprehend the mechanisms and structures involved in toxin routing throughout the nervous system. Thus, multidisciplinary approaches integrating molecular microbiology, membrane biology, biochemistry, physiology, proteomics, and pharmacology will further advance our understanding of the transport mechanisms required for directing molecules to specific locations within the nervous system of eukaryotes. Clearly, specificity of action for the BoNTs has made them very useful therapeutic agents for many human neurological syndromes caused by hyperactivity of cholinergic nerve terminals. It is expected that future pharmacological developments will employ the inherent capabilities of various toxins to deliver biologically-active substances into nerve cells.

## References

[1] Gill D.M., Laskin A.I., Lechevalier H.A. (1987). Bacterial toxins: Lethal amounts. Toxins and Enzymes.

[2] Popoff M.R. (1987). Purification and characterization of *Clostridium sordellii* lethal toxin and cross-reactivity with *Clostridium difficile* cytotoxin. Infect. Immun..

[3] Von Eichel-Streiber C., Harperath U., Bosse D., Hadding U. (1987). Purification of two high molecular weigth toxins of *Clostridium difficile* which are antigenically related. Microb. Pathogen.

[4] Meng X.Q., Kamiya S., Yamakawa K., Ogura H., Nakamura S. (1993). Purification and characterisation of intracellular toxin A of Clostridium difficile. J. Med. Microbiol..

[5] Burleigh D.E., Banks M.R. (2007). Stimulation of intestinal secretion by vasoactive intestinal peptide and cholera toxin. Auton. Neurosci..

[6] Murthy V.N., De Camilli P. (2003). Cell biology of the presynaptic terminal. Annu. Rev. Neurosci..

[7] Takamori S., Holt M., Stenius K., Lemke E.A., Gronborg M., Riedel D., Urlaub H., Schenck S., Brugger B., Ringler P., Muller S.A., Rammner B., Grater F., Hub J.S., De Groot B.L., Mieskes G., Moriyama Y., Klingauf J., Grubmuller H., Heuser J., Wieland F., Jahn R. (2006). Molecular anatomy of a trafficking organelle. Cell.

[8] Kasai H. (1999). Comparative biology of Ca^2+^-dependent exocytosis: implications of kinetic diversity for secretory function. Trends Neurosci..

[9] Jahn R., Scheller R.H. (2006). SNAREs--engines for membrane fusion. Nat. Rev. Mol. Cell. Biol..

[10] Rizo J., Rosenmund C. (2008). Synaptic vesicle fusion. Nat. Struct. Mol. Biol..

[11] Doussau F., Augustine G.J. (2000). The actin cytoskeleton and neurotransmitter release: An overview. Biochimie.

[12] Garner C.C., Kindler S., Gundelfinger E.D. (2000). Molecular determinants of presynaptic active zones. Curr. Opin. Neurobiol..

[13] Petersen O.H. (2003). Localization and regulation of Ca^2+^ entry and exit pathways in exocrine gland cells. Cell Calcium.

[14] Rettig J., Neher E. (2002). Emerging roles of presynaptic proteins in Ca^++^-triggered exocytosis. Science.

[15] Chapman E.R. (2002). Synaptotagmin: A Ca^2+^ sensor that triggers exocytosis?. Nat. Rev. Mol. CellBiol..

[16] Koh T.W., Bellen H.J. (2003). Synaptotagmin I, a Ca^2+^ sensor for neurotransmitter release. Trends Neurosci..

[17] Bhalla A., Chicka M.C., Tucker W.C., Chapman E.R. (2006). Ca(2+)-synaptotagmin directly regulates t-SNARE function during reconstituted membrane fusion. Nat. Struct. Mol. Biol..

[18] Martens S., Kozlov M.M., McMahon H.T. (2007). How synaptotagmin promotes membrane fusion. Science.

[19] Groffen A., Friedrich R., Brian E.C., Ashery U., Verhage M. (2006). DOC2A and DOC2B are sensors for neuronal activity with unique calcium-dependent and kinetic properties. J. Neurochem..

[20] Augustine G.J. (2001). How does calcium trigger neurotransmitter release?. Curr. Opin. Neurobiol..

[21] Segev N. (2001). Ypt/rab gtpases: Regulators of protein trafficking. Sci. STKE.

[22] Eitzen G. (2003). Actin remodeling to facilitate membrane fusion. Biochim. Biophys. Acta.

[23] Bader M.F., Doussau F., Chasserot-Golaz S., Vitale N., Gasman S. (2004). Coupling actin and membrane dynamics during calcium-regulated exocytosis: A role for Rho and ARF GTPases. Biochim. Biophys. Acta.

[24] Hall A. (1998). Rho GTPases and the actin cytoskeleton. Science.

[25] Gasman S., Chasserot-Golaz S., Malacombe M., Way M., Bader M.F. (2004). Regulated exocytosis in neuroendocrine cells: A role for subplasmalemmal Cdc42/N -WASP-induced actin filaments. Mol. Biol. Cell.

[26] Li Q., Ho C.S., Marinescu V., Bhatti H., Bokoch G.M., Ernst S.A., Holz R.W., Stuenkel E.L. (2003). Facilitation of Ca(2+)-dependent exocytosis by Rac1-GTPase in bovine chromaffin cells. J. Physiol..

[27] Doussau F., Gasman S., Humeau Y., Vitiello F., Popoff M.R., Boquet P., Bader M.F., Poulain B. (2000). A Rho-related GTPase is involved in Ca^++^-dependent neurotransmitter exocytosis. J. Biol. Chem..

[28] Humeau Y., Popoff M.R., Kojima H., Dousseau F., Poulain B. (2002). Rac GTPase plays an essential role in exocytosis by controlling the fusion competence in release sites. J. Neurosci..

[29] Momboisse F., Lonchamp E., Calco V., Ceridono M., Vitale N., Bader M.F., Gasman S. (2009). betaPIX-activated Rac1 stimulates the activation of phospholipase D, which is associated with exocytosis in neuroendocrine cells. J. Cell. Sci..

[30] Vitale N., Chasserot-Golaz S., Bailly Y., Morinaga N., Frohman M.A., Bader M.F. (2002). Calcium-regulated exocytosis of dense-core vesicles requires the activation of ADP-ribosylation factor (ARF)6 by ARF nucleotide binding site opener at the plasma membrane. J. Cell. Biol..

[31] Bielinski D.F., Pyun H.Y., Linko-Stentz K., Macara I.G., Fine R.E. (1993). Protein Ral and Rab3a are major GTP-binding proteins of axonal rapid transport and synaptic vesicles and do not redistribute following depolarization stimulated synaptosomal exocytosis. Biochim. Biophys. Acta.

[32] Polzin A., Shipitsin M., Goi T., Feig L.A., Turner T.J. (2002). Ral-GTPase influences the regulation of the readily releasable pool of synaptic vesicles. Mol. Cell Biol..

[33] Luo J.Q., Liu X., Frankel P., Rotunda T., Ramos M., Flom J., Jiang H., Feig L.A., Morris A.J., Kahn R.A., Foster D.A. (1998). Functional association between Arf and RalA in active phospholipase D complex. Proc. Natl. Acad. Sci. USA.

[34] Choi W.S., Kim Y.M., Combs C., Frohman M.A., Beaven M.A. (2002). Phospholipases D1 and D2 regulate different phases of exocytosis in mast cells. J. Immunol..

[35] Humeau Y., Vitale N., Chasserot-Golaz S., Dupont J.L., Du G., Frohman M.A., Bader M.F., Poulain B. (2001). A role for phospholipase D1 in neurotransmitter release. Proc. Natl. Acad. Sci. (USA).

[36] Humeau Y., Doussau F., Popoff M.R., Benfenati F., Poulain B. (2007). Fast changes in the functional status of release sites during short-term plasticity: Involvement of a frequency-dependent bypass of Rac at Aplysia synapses. J. Physiol..

[37] Vitale N., Caumont A.S., Chasserot-Golaz S., Du G., Wu S., Sciorra V.A., Morris A.J., Frohman M.A., Bader M.F. (2001). Phospholipase D1: A key factor for the exocytic machinery in neuroendocrine cells. EMBOJ..

[38] Chernomordik L.V., Kozlov M.M. (2003). Protein-lipid interplay in fusion and fission of biological membranes. Ann. Rev. Biochem..

[39] Bigalke H., Shoer L.F., Aktories K., Just I. (2000). Clostridial neurotoxins. Bacterial Protein Toxins.

[40] Herreros J., Lalli G., Montecucco C., Schiavo G., Alouf J.E., Freer J.H. (1999). Pathophysiological properties of clostridial neurotoxins. The Comprehensive Sourcebook of Bacterial Protein Toxins.

[41] Humeau Y., Doussau F., Grant N.J., Poulain B. (2000). How botulinum and tetanus neurotoxins block neurotransmitter. Biochimie.

[42] Meunier F.A., Herreros J., Schiavo G., Poulain B., Molgo J., Massaro J. (2002). Molecular mechanism of action of botulinal neurotoxins and the synaptic remodeling they induce *in vivo* at the skeletal neuromuscular junction. Handbook of Neurotoxicology.

[43] Meunier F.A., Schiavo G., Molgo J. (2002). Botulinum neurotoxins: From paralysis to recovery of functional neuromuscular trasnmission. J. Physiol..

[44] Poulain B., Popoff M.R., Molgo J. (2008). How do the botulinum neurotoxins block neurotransmitter release: From botulism to the molecular mechanism of action. Botulinum. J..

[45] Schiavo G., Matteoli M., Montecucco C. (2000). Neurotoxins affecting neuroexocytosis. Physiol. Rev..

[46] Hill K.K., Smith T.J., Helma C.H., Ticknor L.O., Foley B.T., Svensson R.T., Brown J.L., Johnson E.A., Smith L.A., Okinaka R.T., Jackson P.J., Marks J.D. (2007). Genetic diversity among Botulinum Neurotoxin-producing clostridial strains. J. Bacteriol..

[47] Smith T.J., Hill K.K., Foley B.T., Detter J.C., Munk A.C., Bruce D.C., Doggett N.A., Smith L.A., Marks J.D., Xie G., Brettin T.S. (2007). Analysis of the neurotoxin complex genes in clostridium botulinum A1-A4 and B1 strains: BoNT/A3, /Ba4 and /B1 clusters are located within plasmids. PLoS ONE.

[48] Smith T.J., Lou J., Geren N., Forsyth M., Tsai R., La Porte S.L., Tepp W.H., Bradshaw M., Johnson E.A., Smith L.A., Marks J.D. (2005). Sequence variation within botulinum neurotoxin serotypes impacts antibody binding and neutralization. Infect. Immun..

[49] Arndt E.R., Jacobson M.J., Abola E.E., Forsyth C.M., Tepp W.H., Marks J.D., Johnson E.A., Stevens E.S. (2006). A structural perspective of the sequence variability within botulinum neurotoxin subtypes A1–A4. J. Mol. Biol..

[50] Chen Y., Korkeala H., Aarnikunnas J., Lindstrom M. (2007). Sequencing the botulinum neurotoxin gene and related genes in Clostridium botulinum type E strains reveals orfx3 and a novel type E neurotoxin subtype. J. Bacteriol..

[51] Carter A.T., Paul C., Mason D.R., Twine S.M., Alston M.J., Logan S.M., Austin J.W., Peck M.W. (2009). Independent evolution of neurotoxin and flagellar genetic loci in proteolytic Clostridium botulinum. BMC Genomics.

[52] Popoff M.R., Marvaud J.C., Alouf J.E., Freer J.H. (1999). Structural and genomic features of clostridial neurotoxins. The Comprehensive Sourcebook of Bacterial Protein Toxins.

[53] Hasegawa K., Watanabe T., Suzuki T., Yamano A., Oikawa T., Sato Y., Kouguchi H., Yoneyama T., Niwa K., Ikeda T., Ohyama T. (2007). A novel subunit structure of clostridium botulinum serotype D toxin complex with three extended arms. J. Biol. Chem..

[54] Lietzow M.A., Gielow E.T., Le D., Zhang J., Verhagen M.F. (2008). Subunit stoichiometry of the Clostridium botulinum type A neurotoxin complex determined using denaturing capillary electrophoresis. Protein J..

[55] Call J.E., Cooke P.H., Miller A.J. (1995). *In situ* characterization of *Clostridium botulinum* neurotoxin synthesis and export. J. Appl. Bacteriol..

[56] Emsley P., Fotinou C., Black I., Fairweather N.F., Charles I.G., Watts C., Hewitt E., Isaacks N.W. (2000). The structures of the Hc fragment of Tetanus Toxin with carbohydrate subunit complexes provide insight into ganglioside binding. J. Biol. Chem..

[57] Lacy D.B., Stevens R.C. (1999). Sequence homology and structural analysis of the clostridial neurotoxins. J. Mol. Biol..

[58] Lacy D.B., Tepp W., Cohen A.C., Das Gupta B.R., Stevens R.C. (1998). Crystal structure of botulinum neurotoxin type A and implications for toxicity. Nat. Struct. Biol..

[59] Umland T.C., Wingert L.M., Swaminathan S., Furey W.F., Schmidt J.J., Sax M. (1997). The structure of the receptor binding fragment H_c_ of tetanus neurotoxin. Nat. Struct. Biol..

[60] Fotinou C., Emsley P., Black I., Ando H., Ishida H., Kiso M., Sinha K.A., Fairweather N.F., Isaacs N.W. (2001). The crystal structure of Tetanus toxin Hc fragment complexed with a synthetic GT1b analogue suggests cross-linking between ganglioside receptors and the toxin. J. Biol. Chem..

[61] Breidenbach M.A., Brunger A.T. (2005). 2.3 A crystal structure of tetanus neurotoxin light chain. Biochemistry.

[62] Fu Z., Chen S., Baldwin M.R., Boldt G.E., Crawford A., Janda K.D., Barbieri J.T., Kim J.J. (2006). Light chain of botulinum neurotoxin serotype A: Structural resolution of a catalytic intermediate. Biochemistry.

[63] Swaminathan S., Eswaramoorthy S. (2000). Structural analysis of the catalytic and binding sites of *Clostridium botulinum* neurotoxin B. Nat. Struct. Biol..

[64] Stenmark P., Dupuy J., Imamura A., Kiso M., Stevens R.C. (2008). Crystal structure of botulinum neurotoxin type A in complex with the cell surface co-receptor GT1b-insight into the toxin-neuron interaction. PLoS Pathogen.

[65] Kumaran D., Eswaramoorthy S., Furey W., Navaza J., Sax M., Swaminathan S. (2009). Domain organization in Clostridium botulinum neurotoxin type E is unique: Its implication in faster translocation. J. Mol. Biol..

[66] Maksymowych A.B., Simpson L.L. (1998). Binding and transcytosis of botulinum neurotoxin by polarized human carcinoma cells. J. Biol. Chem..

[67] Maksymowych A.B., Simpson L.I. (2004). Structural features of the botulinum neurotoxin molecule that govern binding and transcytosis across polarized human intestinal epithelial cells. J. Pharmacol. Exp. Ther..

[68] Ahsan C.R., Hajnoczky G., Maksymowych A.B., Simpson L.L. (2005). Visualization of binding and transcytosis of botulinum toxin by human intestinal epithelial cells. J. Pharmacol. Exp. Ther..

[69] Couesnon A., Pereira Y., Popoff M.R. (2008). Receptor-mediated transcytosis of botulinum neurotoxin A through intestinal cell monolayers. Cell Microbiol..

[70] Matsumura T., Jin Y., Kabumoto Y., Takegahara Y., Oguma K., Lencer W.I., Fujinaga Y. (2007). The HA proteins of botulinum toxin disrupt intestinal epithelial intercellular junctions to increase toxin absorption. Cel Microbiol..

[71] Jin Y., Takegahara Y., Sugawara Y., Matsumura T., Fujinaga Y. (2009). Disruption of the epithelial barrier by botulinum haemagglutinin (HA) proteins—differences in cell tropism and the mechanism of action between HA proteins of types A or B, and HA proteins of type C. Microbiology.

[72] Wellhöner H.H., Simpson L.L. (1989). Clostridial toxins and the central nervous system: Studies on *in situ* tissues. Botulinum Neurotoxin and Tetanus Toxin.

[73] Manning K.A., Erichsen J.T., Evinger C. (1990). Retrograde transneuronal transport properties of fragment C of tetanus toxin. Neuroscience.

[74] Rossetto O., Seveso M., Caccin P., Schiavo G., Montecucco C. (2001). Tetanus and botulinum neurotoxins: Turning bad guys into good by research. Toxicon.

[75] Dong M., Liu H., Tepp W.H., Johnson E.A., Janz R., Chapman E.R. (2008). Glycosylated SV2A and SV2B mediate the entry of botulinum neurotoxin E into neurons. Mol. Biol. Cell..

[76] Dong M., Tepp W.H., Liu H., Johnson E.A., Chapman E.R. (2007). Mechanism of botulinum neurotoxin B and G entry into hippocampal neurons. J. Cell. Biol..

[77] Dong M., Yeh F., Tepp W.H., Dean C., Johnson E.A., Janz R., Chapman E.R. (2006). SV2 is the protein receptor for botulinum neurotoxin A. Science.

[78] Mahrhold S., Rummel A., Bigalke H., Davletov B., Binz T. (2006). The synaptic vesicle protein 2C mediates the uptake of botulinum neurotoxin A into phrenic nerves. FEBS Lett..

[79] Nishiki T., Kamata Y., Nemoto Y., Omori A., Ito T., Takahashi M., Kozaki S. (1994). Identification of protein receptor for *Clostridium botulinum* type B neurotoxin in rat brain synaptosomes. J. Biol. Chem..

[80] Rummel A., Hafner K., Mahrhold S., Darashchonak N., Holt M., Jahn R., Beermann S., Karnath T., Bigalke H., Binz T. (2009). Botulinum neurotoxins C, E and F bind gangliosides *via* a conserved binding site prior to stimulation-dependent uptake with botulinum neurotoxin F utilising the three isoforms of SV2 as second receptor. J. Neurochem..

[81] Rummel A., Karnath T., Henke T., Bigalke H., Binz T. (2004). Synaptotagmins I and II act as nerve cell receptors for botulinum neurotoxin G. J. Biol. Chem..

[82] Herreros J., Ng T., Schiavo G. (2001). Lipid rafts act as specialized domains for tetanus toxin binding and internalization into neurons. Mol. Biol. Cell.

[83] Munro P., Kojima H., Dupont J.L., Bossu J.L., Poulain B., Boquet P. (2001). High sensitivity of mouse neuronal cells to tetanus toxin requires a GPI-anchored protein. Biochem. Biophys. Res. Comm..

[84] Rummel A., Bade S., Alves J., Bigalke H., Binz T. (2003). Two carbohydrate binding sites in the H_cc_-domain of tetanus neurotoxin are required for toxicity. J. Mol. Biol..

[85] Rummel A., Eichner T., Weil T., Karnath T., Gutcaits A., Mahrhold S., Sandhoff K., Proia R.L., Acharya K.R., Bigalke H., Binz T. (2007). Identification of the protein receptor binding site of botulinum neurotoxins B and G proves the double-receptor concept. Proc. Natl. Acad. Sci. USA.

[86] Rummel A., Mahrhold S., Bigalke H., Binz T. (2004). The H_cc_-domain of botulinum neurotoxins A and B exhibits a singular gangliosside binding site displaying serotype specific carbohydrate interaction. Mol. Microbiol..

[87] Chen C., Fu Z., Kim J.J., Barbieri J.T., Baldwin M.R. (2009). Gangliosides as high affinity receptors for tetanus neurotoxin. J. Biol. Chem..

[88] Tsukamoto K., Kozai Y., Ihara H., Kohda T., Mukamoto M., Tsuji T., Kozaki S. (2008). Identification of the receptor-binding sites in the carboxyl-terminal half of the heavy chain of botulinum neurotoxin types C and D. Microb. Pathog..

[89] Muraro L., Tosatto S., Motterlini L., Rossetto O., Montecucco C. (2009). The N-terminal half of the receptor domain of botulinum neurotoxin A binds to microdomains of the plasma membrane. Biochem. Biophys. Res. Commun..

[90] Yowler B.C., Schengrund C.L. (2004). Botulinum neurotoxin A changes conformation upon binding to ganglioside GT1b. Biochemistry.

[91] Chen C., Baldwin M.R., Barbieri J.T. (2008). Molecular basis for tetanus toxin coreceptor interactions. Biochemistry.

[92] Burgen A.S., Dickens F., Zatman L.J. (1949). The action of botulinum toxin on the neuro-muscular junction. J. Physiol..

[93] Dunant Y., Esquerda J.E., Loctin F., Marsal J., Muller D. (1987). Botulinum toxin inhibits quantal acetylcholine release and energy metabolism in the Torpedo electric organ. J. Physiol..

[94] Poulain B., Tauc L., Maisey E.A., Wadsworth J.D., Mohan P.M., Dolly J.O. (1988). Neurotransmitter release is blocked intracellularly by botulinum neurotoxin, and this requires uptake of both toxin polypeptides by a process mediated by the larger chain. Proc. Natl. Acad. Sci. USA.

[95] Sanchez-Prieto J., Sihra T.S., Evans D., Ashton A., Dolly J.O., Nicholls D.G. (1987). Botulinum toxin A blocks glutamate exocytosis from guinea-pig cerebral cortical synaptosomes. Eur. J. Biochem..

[96] Cui M., Khanijou S., Rubino J., Aoki K.R. (2004). Subcutaneous administration of botulinum toxin A reduces formalin-induced pain. Pain.

[97] Foran P.G., Mohammed N., Lisk G.O., Nagwaney S., Lawrence G.W., Johnson E., Smith L., Aoki K.R., Dolly O.J. (2003). Evaluation of the therapeutic usefulness of botulinum neurotoxin B, C1, E and F compared with the long lasting type A. J. Biol. Chem..

[98] Khairallah G., Andreoletti J.B., Jover E., Simon E. (2008). Measurement of botulinum toxin activity: Towards a new cellular culture assay?. Ann. Chir. Plast. Esthet..

[99] McMahon H.T., Foran P., Dolly J.O., Verhage M., Wiegant V.M., Nicholls D.G. (1992). Tetanus toxin and botulinum toxins type A and B inhibit glutamate, gamma-aminobutyric acid, aspartate, and met-enkephalin release from synaptosomes. Clues to the locus of action. J. Biol. Chem..

[100] Ashton A.C., Dolly J.O. (1988). Characterization of the inhibitory action of botulinum neurotoxin type A on the release of several transmitters from rat cerebrocortical synaptosomes. J. Neurochem..

[101] Neale E.A., Bowers L.M., Jia M., Bateman K.E., Williamson L.C. (1999). Botulinum neurotoxin A blocks synaptic vesicle exocytosis but not endocytosis at the nerve terminal. J. Cell. Biol..

[102] Maisey E.A., Wadsworth J.D., Poulain B., Shone C.C., Melling J., Gibbs P., Tauc L., Dolly J.O. (1988). Involvement of the constituent chains of botulinum neurotoxins A and B in the blockade of neurotransmitter release. Eur. J. Biochem..

[103] Najib A., Pelliccioni P., Gil C., Aguilera J. (1999). Clostridium neurotoxins influence serotonin uptake and release differently in rat brain synaptosomes. J. Neurochem..

[104] Marsal J., Egea G., Solsona C., Rabasseda X., Blasi J. (1989). Botulinum toxin type A blocks the morphological changes induced by chemical stimulation on the presynaptic membrane of Torpedo synaptosomes. Proc. Natl. Acad. Sci. USA.

[105] Khera M., Somogyi G.T., Kiss S., Boone T.B., Smith C.P. (2004). Botulinum toxin A inhibits ATP release from bladder urothelium after chronic spinal cord injury. Neurochem. Int..

[106] Smith C.P., Vemulakonda V.M., Kiss S., Boone T.B., Somogyi G.T. (2005). Enhanced ATP release from rat bladder urothelium during chronic bladder inflammation: Effect of botulinum toxin A. Neurochem. Int..

[107] Tompkins J.D., Parsons R.L. (2006). Exocytotic release of ATP and activation of P2X receptors in dissociated guinea pig stellate neurons. Am. J. Physiol. Cell Physiol..

[108] Smyth L.M., Breen L.T., Mutafova-Yambolieva V.N. (2006). Nicotinamide adenine dinucleotide is released from sympathetic nerve terminals *via* a botulinum neurotoxin A-mediated mechanism in canine mesenteric artery. Am. J. Physiol. Heart. Circ. Physiol..

[109] Breen L.T., Smyth L.M., Yamboliev I.A., Mutafova-Yambolieva V.N. (2006). beta-NAD is a novel nucleotide released on stimulation of nerve terminals in human urinary bladder detrusor muscle. Am. J. Physiol. Renal. Physiol..

[110] Welch M.J., Purkiss J.R., Foster K.A. (2000). Sensitivity of embryonic rat dorsal root ganglia neurons to Clostridium botulinum neurotoxins. Toxicon.

[111] Duggan M.J., Quinn C.P., Chaddock J.A., Purkiss J.R., Alexander F.C., Doward S., Fooks S.J., Friis L.M., Hall Y.H., Kirby E.R., Leeds N., Moulsdale H.J., Dickenson A., Green G.M., Rahman W., Suzuki R., Shone C.C., Foster K.A. (2002). Inhibition of release of neurotransmitters from rat dorsal root ganglia by a novel conjugate of a Clostridium botulinum toxin A endopeptidase fragment and Erythrina cristagalli lectin. J. Biol. Chem..

[112] Durham P.L., Cady R., Cady R. (2004). Regulation of calcitonin gene-related peptide secretion from trigeminal nerve cells by botulinum toxin type A: Implications for migraine therapy. Headache: J. Head and Face Pain.

[113] Rapp D.E., Turk K.W., Bales G.T., Cook S.P. (2006). Botulinum toxin type an inhibits calcitonin gene-related peptide release from isolated rat bladder. J. Urol..

[114] Hassan S.M., Jennekens F.G., Wieneke G., Veldman H. (1994). Calcitonin gene-related peptide-like immunoreactivity, in botulinum toxin-paralysed rat muscles. Neuromuscul. Disord..

[115] Meunier F.A., Colasante C., Faille L., Gastard M., Molgo J. (1996). Upregulation of calcitonin gene-related peptide at mouse motor nerve terminals poisoned with botulinum type-A toxin. Pflugers. Arch..

[116] Sala C., Andreose J.S., Fumagalli G., Lomo T. (1995). Calcitonin gene-related peptide: Possible role in formation and maintenance of neuromuscular junctions. J. Neurosci..

[117] Tarabal O., Caldero J., Ribera J., Sorribas A., Lopez R., Molgo J., Esquerda J.E. (1996). Regulation of motoneuronal calcitonin gene-related peptide (CGRP) during axonal growth and neuromuscular synaptic plasticity induced by botulinum toxin in rats. Eur. J. Neurosci..

[118] Swartling C., Naver H., Pihl-Lundin I., Hagforsen E., Vahlquist A. (2004). Sweat gland morphology and periglandular innervation in essential palmar hyperhidrosis before and after treatment with intradermal botulinum toxin. J. Am. Acad. Dermatol..

[119] Morris J.L., Jobling P., Gibbins I.L. (2002). Botulinum neurotoxin A attenuates release of norepinephrine but not NPY from vasoconstrictor neurons. Am. J. Physiol. Heart. Circ. Physiol..

[120] Jones O.M., Brading A.F., Mortensen N.J. (2004). Mechanism of action of botulinum toxin on the internal anal sphincter. Br. J. Surg..

[121] Moffatt J.D., Cocks T.M., Page C.P. (2004). Role of the epithelium and acetylcholine in mediating the contraction to 5-hydroxytryptamine in the mouse isolated trachea. Br. J. Pharmacol..

[122] Verderio C., Pozzi D., Pravettoni E., Inverardi F., Schenk U., Coco S., Proux-Gillardeaux V., Galli T., Rossetto O., Frassoni C., Matteoli M. (2004). SNAP-25 modulation of calcium dynamics underlies differences in GABAergic and glutamatergic responsiveness to depolarization. Neuron.

[123] Penner R., Neher E., Dreyer F. (1986). Intracellularly injected tetanus toxin inhibits exocytosis in bovine adrenal chromaffin cells. Nature.

[124] Ahnert-Hilger G., Bader M.F., Bhakdi S., Gratzl M. (1989). Introduction of macromolecules into bovine adrenal medullary chromaffin cells and rat pheochromocytoma cells (PC12) by permeabilization with streptolysin O: Inhibitory effect of tetanus toxin on catecholamine secretion. J. Neurochem..

[125] Ahnert-Hilger G., Weller U., Dauzenroth M.E., Habermann E., Gratzl M. (1989). The tetanus toxin light chain inhibits exocytosis. FEBS Lett..

[126] Abdipranoto A., Liu G.J., Werry E.L., Bennett M.R. (2003). Mechanisms of secretion of ATP from cortical astrocytes triggered by uridine triphosphate. Neuroreport.

[127] Araque A., Li N., Doyle R.T., Haydon P.G. (2000). SNARE protein-dependent glutamate release from astrocytes. J. Neurosci..

[128] Verderio C., Coco S., Rossetto O., Montecucco C., Matteoli M. (1999). Internalization and proteolytic action of botulinum toxins in CNS neurons and astrocytes. J. Neurochem..

[129] Regazzi R., Sadoul K., Meda P., Kelly R.B., Halban P.A., Wollheim C.B. (1996). Mutational analysis of VAMP domains implicated in Ca^2+^-induced insulin exocytosis. EMBO J..

[130] Rosado J.A., Redondo P.C., Salido G.M., Sage S.O., Pariente J.A. (2005). Cleavage of SNAP-25 and VAMP-2 impairs store-operated Ca^2+^ entry in mouse pancreatic acinar cells. Am. J. Physiol. Cell. Physiol..

[131] Redondo P.C., Harper A.G., Salido G.M., Pariente J.A., Sage S.O., Rosado J.A. (2004). A role for SNAP-25 but not VAMPs in store-mediated Ca^2+^ entry in human platelets. J. Physiol..

[132] Semba T., Kano M. (1969). Glycine in the spinal cord of cats with local tetanus rigidity. Science.

[133] Fedinec A.A., Shank R.P. (1971). Effect of tetanus toxin on the content of glycine, gamma-aminobutyric acid, glutamate, glutamine and aspartate in the rat spinal cord. J. Neurochem..

[134] Williamson L.C., Fitzgerald S.C., Neale E.A. (1992). Differential effects of tetanus toxin on inhibitory and excitatory neurotransmitter release from mammalian spinal cord cells in culture. J. Neurochem..

[135] Habermann E. (1988). Inhibition by tetanus and botulinum A toxin of the release of [3H]noradrenaline and [3H]GABA from rat brain homogenate. Experientia.

[136] Collingridge G.L., Davies J. (1982). Reversible effects of tetanus toxin on striatal-evoked responses and [3H]-gamma-aminobutyric acid release in the rat substantia nigra. Br. J. Pharmacol..

[137] Collingridge G.L., Thompson P.A., Davies J., Mellanby J. (1981). *In vitro* effect of tetanus toxin on GABA release form rat hippocampal slices. J. Neurochem..

[138] Albus U., Habermann E. (1983). Tetanus toxin inhibits the evoked outflow of an inhibitory (GABA) and an excitatory (D-aspartate) amino acid from particulate brain cortex. Toxicon.

[139] Pearce B.R., Gard A.L., Dutton G.R. (1983). Tetanus toxin inhibition of K+-stimulated [3H]GABA release from developing cell cultures of the rat cerebellum. J. Neurochem..

[140] Van Vliet B.J., Sebben M., Dumuis A., Gabrion J., Bockaert J., Pin J.P. (1989). Endogenous amino acid release from cultured cerebellar neuronal cells: effect of tetanus toxin on glutamate release. J. Neurochem..

[141] Bagetta G., Nistico G. (1992). Glutamate transmission is involved in the mechanisms of neuronal degeneration produced by intrahippocampal tetanus toxin in rats. Toxicol. Lett..

[142] Bradford S.E., Nadler J.V. (2004). Aspartate release from rat hippocampal synaptosomes. Neuroscience.

[143] Lu W., Man H., Ju W., Trimble W.S., MacDonald J.F., Wang Y.T. (2001). Activation of synaptic NMDA receptors induces membrane insertion of new AMPA receptors and LTP in cultured hippocampal neurons. Neuron.

[144] Lindlbauer R., Mohrmann R., Hatt H., Gottmann K. (1998). Regulation of kinetic and pharmacological properties of synaptic NMDA receptors depends on presynaptic exocytosis in rat hippocampal neurones. J. Physiol..

[145] Fleck M.W., Barrionuevo G., Palmer A.M. (2001). Release of D, L-threo-beta-hydroxyaspartate as a false transmitter from excitatory amino acid-releasing nerve terminals. Neurochem. Int..

[146] Habermann E., Muller H., Hudel M. (1988). Tetanus toxin and botulinum A and C neurotoxins inhibit noradrenaline release from cultured mouse brain. J. Neurochem..

[147] Figliomeni B., Grasso A. (1985). Tetanus toxin affects the K+-stimulated release of catecholamines from nerve growth factor-treated PC12 cells. Biochem. Biophys. Res. Commun..

[148] Bansal M.K., Phillips J.H., van Heyningen S. (1990). The inhibition by pertussis and tetanus toxins of evoked catecholamine release from intact and permeabilized bovine adrenal chromaffin cells. FEBS Lett..

[149] Stecher B., Hens J., Weller U., Gratzl M., Gispen W.H., De Graan P.N. (1992). Noradrenaline release from permeabilized synaptosomes is inhibited by the light chain of tetanus toxin. FEBS Lett..

[150] Ashton A.C., Dolly J.O. (1997). Microtubules and microfilaments participate in the inhibition of synaptosomal noradrenaline release by tetanus toxin. J. Neurochem..

[151] Tuz K., Pasantes-Morales H. (2005). Hyposmolarity evokes norepinephrine efflux from synaptosomes by a depolarization- and Ca^2+^-dependent exocytotic mechanism. Eur. J. Neurosci..

[152] Britton P., Whitton P.S., Bowery N.G. (1995). Effect of tetanus toxin on basal and evoked release of 5-hydroxytryptamine and dopamine in rat hippocampus *in vivo*. Brain Res..

[153] Whitton P.S., Britton P., Bowery N.G. (1992). Tetanus toxin alters 5-hydroxytryptamine, dopamine, and their metabolites in rat hippocampus measured by *in vivo* microdialysis. Neurosci. Lett..

[154] Gobbi M., Facchiano F., Frittoli E., Luini A., Mennini T. (1993). Tetanus toxin inhibits depolarization-induced [3H]serotonin release from rat brain cortex synaptosomes. Neurosci. Lett..

[155] Inserte J., Najib A., Pelliccioni P., Gil C., Aguilera J. (1999). Inhibition by tetanus toxin of sodium-dependent, high-affinity [3H]5-hydroxytryptamine uptake in rat synaptosomes. Biochem. Pharmacol..

[156] Gil C., Najib A., Aguilera J. (2003). Serotonin transport is modulated differently by tetanus toxin and growth factors. Neurochem. Int..

[157] Pelliccioni P., Gil C., Najib A., Sarri E., Picatoste F., Aguilera J. (2001). Tetanus toxin modulates serotonin transport in rat-brain neuronal cultures. J. Mol. Neurosci..

[158] Najib A., Pelliccioni P., Gil C., Aguilera J. (2000). Serotonin transporter phosphorylation modulated by tetanus toxin. FEBS Lett..

[159] Sandberg K., Berry C.J., Eugster E., Rogers T.B. (1989). A role for cGMP during tetanus toxin blockade of acetylcholine release in the rat pheochromocytoma (PC12) cell line. J. Neurosci..

[160] Sandberg K., Berry C.J., Rogers T.B. (1989). Studies on the intoxication pathway of tetanus toxin in the rat pheochromocytoma (PC12) cell line. Binding, internalization, and inhibition of acetylcholine release. J. Biol. Chem..

[161] Egea G., Rabasseda X., Solsona C., Marsal J., Bizzini B. (1990). Tetanus toxin blocks potassium-induced transmitter release and rearrangement of intramembrane particles at pure cholinergic synaptosomes. Toxicon.

[162] Bigalke H., Dimpfel W., Habermann E. (1978). Suppression of 3H-acetylcholine release from primary nerve cell cultures by tetanus and botulinum-A toxin. Naunyn. Schmiedebergs. Arch. Pharmacol..

[163] Mochida S., Poulain B., Weller U., Habermann E., Tauc L. (1989). Light chain of tetanus toxin intracellularly inhibits acetylcholine release at neuro-neuronal synapses, and its internalization is mediated by heavy chain. FEBS Lett..

[164] Kang N., Xu J., Xu Q., Nedergaard M., Kang J. (2005). Astrocytic glutamate release-induced transient depolarization and epileptiform discharges in hippocampal CA1 pyramidal neurons. J. Neurophysiol..

[165] Galli T., Chilcote T., Mundigl O., Binz T., Niemann H., De Camilli P. (1994). Tetanus toxin-mediated cleavage of cellubrevin impairs exocytosis of transferrin receptor-containing vesicles in CHO cells. J. Cell. Biol..

[166] Lalli G., Bohnert S., Deinhardt K., Verastegui C., Schiavo G. (2003). The journey of tetanus and botulinum neurotoxins in neurons. Trends Microbiol..

[167] Lalli G., Schiavo G. (2002). Analysis of retrograde transport in motor neurons reveals common endocytic carriers for tetanus toxin and neutrophin receptor p75^NTR^. J. Cell Biol..

[168] Bohnert S., Deinhardt K., Salinas S., Schiavo G., Alouf J.E., Popoff M.R. (2006). Uptake and transport of clostridium neurotoxins. The Sourcebook of Comprehensive Bacterial Protein Toxins.

[169] Bohnert S., Schiavo G. (2005). Tetanus toxin is transported in a novel neuronal compartment characterized by a specialized pH regulation. J. Biol. Chem..

[170] Deinhardt K., Berminghausen O., Willison H.J., Hopkins C.R., Schiavo G. (2006). Tetanus toxin is internalized by a sequential clathrin-dependent mechanism initiated within lipid microdomains and independent of epsin1. J. Cell. Biol..

[171] Deinhardt K., Salinas S., Verastegui C., Watson R., Worth D., Hanrahan S., Bucci C., Schiavo G. (2006). Rab5 and Rab7 control endocytic sorting along the axonal retrograde transport pathway. Neuron.

[172] Li Y., Foran P., Lawrence G., Mohammed N., Chan-Kwo-Chion C., Lisk G., Aoki R., Dolly O. (2001). Recombinant forms of tetanus toxin engineered for examining and exploiting neuronal trafficking pathways. J. Biol. Chem..

[173] Maskos U., Kissa K., St Cloment C., Brulet P. (2002). Retrograde trans-synaptic transfer of green fluorescent protein allows the genetic mapping of neuronal circuits in transgenic mice. Proc. Natl. Acad.Sci. USA.

[174] Galloux M., Vitrac H., Montagner C., Raffestin S., Popoff M.R., Chenal A., Forge V., Gillet D. (2008). Membrane Interaction of botulinum neurotoxin A translocation (T) domain. The belt region is a regulatory loop for membrane interaction. J. Biol. Chem..

[175] Koriazova L.K., Montal M. (2003). Translocation of botulinum neurotoxin light chain protease through the heavy chain channel. Nat. Struct. Biol..

[176] Fischer A., Montal M. (2007). Crucial role of the disulfide bridge between botulinum neurotoxin light and heavy chains in protease translocation across membranes. J. Biol. Chem..

[177] Montal M. (2009). Translocation of botulinum neurotoxin light chain protease by the heavy chain protein-conducting channel. Toxicon.

[178] Ratts R., Trujillo C., Bharti A., vanderSpek J., Harrison R., Murphy J.R. (2005). A conserved motif in transmembrane helix 1 of diphtheria toxin mediates catalytic domain delivery to the cytosol. Proc. Natl. Acad. Sci. USA.

[179] Tucker W.C., Weber T., Chapman E.R. (2004). Reconstitution of Ca^2+^-regulated membrane fusion by synaptotagmin and SNAREs. Science.

[180] Sakaba T., Stein A., Jahn R., Neher E. (2005). Distinct kinetic changes in neurotransmitter release after SNARE protein cleavage. Science.

[181] Lynch K.L., Gerona R.R., Kielar D.M., Martens S., McMahon H.T., Martin T.F. (2008). Synaptotagmin-1 utilizes membrane bending and SNARE binding to drive fusion pore expansion. Mol. Biol. Cell..

[182] Gerona R.R., Larsen E.C., Kowalchyk J.A., Martin T.F. (2000). The C terminus of SNAP25 is essential for Ca(2+)-dependent binding of synaptotagmin to SNARE complexes. J. Biol. Chem..

[183] Apland J.P., Adler M., Oyler G.A. (2003). Inhibition of neurotransmitter release by peptides that mimic the N-terminal domain of SNAP-25. J. Protein. Chem..

[184] Gutierrez R., Garcia T., Gonzalez I., Sanz B., Hernandez P.E., Martin R. (1997). A quantitative PCR-ELISA for the rapid enumeration of bacteria in refrigerated raw milk. J. Appl. MIcrobiol..

[185] Keller J.E., Neale E.A. (2001). The role of the synaptic protein snap-25 in the potency of botulinum neurotoxin type A. J. Biol. Chem..

[186] Chen Y.A., Scales S.J., Jagath J.R., Scheller R.H. (2001). A discontinuous SNAP-25 C-terminal coil supports exocytosis. J. Biol. Chem..

[187] Chen Y.A., Scales S.J., Patel S.M., Doung Y.C., Scheller R.H. (1999). SNARE complex formation is triggered by Ca^2+^ and drives membrane fusion. Cell.

[188] Schuette C.G., Hatsuzawa K., Margittai M., Stein A., Riedel D., Kuster P., Konig M., Seidel C., Jahn R. (2004). Determinants of liposome fusion mediated by synaptic SNARE proteins. Proc. Natl. Acad. Sci. USA.

[189] Bajohrs M., Rickman C., Binz T., Davletov B. (2004). A molecular basis underlying differences in the toxicity of botulinum serotypes A and E. EMBO Rep..

[190] Salem N., Faundez V., Horng J.T., Kelly R.B. (1998). A v-SNARE participates in synaptic vesicle formation mediated by the AP3 adaptor complex. Nat. Neurosci..

[191] Cornille F., Deloye F., Fournie-Zaluski M.C., Roques B.P., Poulain B. (1995). Inhibition of neurotransmitter release by synthetic proline-rich peptides shows that the N-terminal domain of vesicle-associated membrane protein/synaptobrvin is critical for neuro-exocytosis. J. Biol. Chem..

[192] Foran P., Lawrence G.W., Shone C.C., Foster K.A., Dolly J.O. (1996). Botulinum neurotoxin C1 cleaves both syntaxin and SNAP-25 in intact and permeabilized chro-maffin cells: Correlation with its blockade of catecholamine release. Biochemistry.

[193] Vaidyanathan V.V., Yoshino K., Jahnz M., Dorries C., Bade S., Nauenburg S., Niemann H., Binz T. (1999). Proteolysis of SNAP-25 isoforms by botulinum neurotoxin types A, C, and E: Domains and amino acid residues controlling the formation of enzyme-substrate complexes and cleavage. J. Neurochem..

[194] O'Connor V., Heuss C., De Bello W.M., Dresbach T., Charlton M.P., Hunt J.H., Pellegrini L.L., Hodel A., Burger M.M., Betz H., Augustine G.J., Schafer T. (1997). Disruption of syntaxin-mediated protein interactions blocks neurotransmitter secretion. Proc. Natl. Acad. Sci. USA.

[195] Capogna M., McKinney R.A., O'Connor V., Gahwiler B.H., Thompson S.M. (1997). Ca^2+^ or Sr^2+^ partially rescues synaptic transmission in hippocampal cultures treated with botulinum toxin A and C, but not tetanus toxin. J. Neurosci..

[196] Williamson L.C., Halpern J.L., Montecucco C., Brown J.E., Neale E.A. (1996). Clostridial neurotoxins and substrate proteolysis in intact neurons: Botulinum neurotoxin C acts on synaptosomal-associated protein of 25 kDa. J. Biol. Chem..

[197] Poulain B., Stiles B.G., Popoff M.R., Molgo J., Alouf J.E., Popoff M.R. (2006). Attack of the nervous system by clostridial toxins: Physical findings, cellular and molecular actions. The Sourcebook of Bacterial Protein Toxins.

[198] Keller J.E., Neale E.A., Oyler G., Adler M. (1999). Persistence of botulinum neurotoxin action in cultured spinal cord cells. FEBS Lett..

[199] O'Sullivan G.A., Mohammed N., Foran P.G., Lawrence G.W., Dolly J.O. (1999). Rescue of exocytosis in botulinum toxin A-poisoned chromaffin cells by expression of cleavage-resistant SNAP-25. J. Biol. Chem..

[200] Fernandez-Salas E., Steward L.E., Ho H., Garay P.E., Sun S.W., Gilmore M.A., Ordas J.V., Wang J., Francis J., Aoki K.R. (2004). Plasma membrane localization signals in the light chain of botulinum neurotoxin. Proc. Natl. Acad. Sci. USA.

[201] Hayashi T., McMahon H., Yamashi S., Binz T., Hata Y., Südhof T.C., Niemann H. (1994). Synaptic vesicle membrane fusion complex: action of clostridial neurotoxins on assembly. EMBO J..

[202] Pellegrini L.L., O'Connor V., Lottspeich F., Betz H. (1995). Clostridial neurotoxins compromise the stability of a low energy SNARE complex mediating NSF activation of synaptic vesicle fusion. EMBO J..

[203] Cohen R., Atlas D. (2004). R-type voltage-gated Ca(2+) channel interacts with synaptic proteins and recruits synaptotagmin to the plasma membrane of Xenopus oocytes. Neuroscience.

[204] Degtiar V.E., Scheller R.H., Tsien R.W. (2000). Syntaxin modulation of slow inactivation of N-type calcium channels. J. Neurochem..

[205] Stanley E.F. (2003). Syntaxin I modulation of presynaptic calcium channel inactivation revealed by botulinum toxin C1. Eur. J. Neurosci..

[206] Wiser O., Trus M., Hernandez A., Renstrom E., Barg S., Rorsman P., Atlas D. (1999). The voltage sensitive Lc-type Ca^2+^ channel is functionally coupled to the exocytotic machinery. Proc. Natl. Acad. Sci. USA.

[207] Bergsman J.B., Tsien R.W. (2000). Syntaxin modulation of calcium channels in cortical synaptosomes as revealed by botulinum toxin C1. J. Neurosci..

[208] Aleu J., Blasi J., Solsona C., Marsal J. (2002). Calcium-dependent acetylcholine release from Xenopus oocytes: Simultaneous ionic currents and acetylcholine release recordings. Eur. J. Neurochem..

[209] Stanley E.F., Mirotznik R.R. (1997). Cleavage of syntaxin prevents G-protein regulation of presynaptic calcium channels. Nature.

[210] Ji J., Tsuk S., Salapatek A.M., Huang X., Chikvashvili D., Pasyk E.A., Kang Y., Sheu L., Tsushima R., Diamant N., Trimble W.S., Lotan I., Gaisano H.Y. (2002). The 25-kDa synaptosome-associated protein (SNAP-25) binds and inhibits delayed rectifier potassium channels in secretory cells. J. Biol. Chem..

[211] Putney J.W. (1986). A model for receptor-regulated calcium entry. Cell Calcium.

[212] Lewis R.S. (2007). The molecular choreography of a store-operated calcium channel. Nature.

[213] Yao Y., Ferrer-Montiel A.V., Montal M., Tsien R.Y. (1999). Activation of store-operated Ca^2+^ current in Xenopus oocytes requires SNAP-25 but not a diffusible messenger. Cell.

[214] Alderton J.M., Ahmed S.A., Smith L.A., Steinhardt R.A. (2000). Evidence for a vesicle-mediated maintenance of store-operated calcium channels in a human embryonic kidney cell line. Cell Calcium.

[215] Rosado J.A., Redondo P.C., Sage S.O., Pariente J.A., Salido G.M. (2005). Store-operated Ca^2+^ entry: Vesicle fusion or reversible trafficking and de novo conformational coupling?. J. Cell Physiol..

[216] Woodard G.E., Salido G.M., Rosado J.A. (2008). Enhanced exocytotic-like insertion of Orai1 into the plasma membrane upon intracellular Ca^2+^ store depletion. Am. J. Physiol. Cell Physiol..

[217] Fili O., Michaelevski I., Bledi Y., Chikvashvili D., Singer-Lahat D., Boshwitz H., Linial M., Lotan I. (2001). Direct interaction of a brain voltage-gated K^+^ channel with syntaxin 1A: Functional impact on channel gating. J. Neurosci..

[218] Michaelevski I., Chikvashvili D., Tsuk S., Singer-Lahat D., Kang Y., Linial M., Gaisano H.Y., Fili O., Lotan I. (2003). Direct interaction of target SNAREs with the Kv2.1 channel. Modal regulation of channel activation and inactivation gating. J. Biol. Chem..

[219] Tsuk S., Michaelevski I., Bentley G.N., Joho R.H., Chikvashvili D., Lotan I. (2005). Kv2.1 channel activation and inactivation is influenced by physical interactions of both syntaxin 1A and the syntaxin 1A/soluble N-ethylmaleimide-sensitive factor-25 (t-SNARE) complex with the C terminus of the channel. Mol. Pharmacol..

[220] Rossetto O., Morbiato L., Caccin P., Rigoni M., Montecucco C. (2006). Presynaptic enzymatic neurotoxins. J. Neurochem..

[221] Li Y., Foran P., Fairweather N.F., de Paiva A., Weller U., Dougan G., Dolly J.O. (1994). A single mutation in the recombinant light chain of tetanus toxin abolishes its proteolytic activity and removes the toxicity seen after reconstitution with native heavy chain. Biochemistry.

[222] Yamasaki S., Baumeister A., Binz T., Blasi J., Link E., Cornille F., Roques B., Fykse E.M., Südhof T.C., Jahn R., Niemann H. (1994). Cleavage of members of the synaptobrevin/VAMP family by types D and F botulinal neurotoxins and tetanus toxin. J. Biol. Chem..

[223] Ashton A.C., Li Y., Doussau F., Weller U., Dougan G., Poulain B., Dolly O. (1995). Tetanus toxin inhibits neuroexocytosis even when its Zn^2+^-dependent proteasea ctivity is removed. J. Biol. Chem..

[224] Niemann H., Blasi J., Jahn R. (1994). Clostridial neurotoxins: New tools for dissecting exocytosis. Trends Cell Biol..

[225] De Paiva A., Ashton A.C., Foran P., Schiavo G., Montecucco C., Dolly J.O. (1993). Botulinum A like type B and tetanus toxins fulfils criteria for being a zinc-dependent protease. J. Neurochem..

[226] Cenci Di Bello I., Poulain B., Shone C.C., Tauc L., Dolly J.O. (1994). Antagonism of the intracellular action of botulinum neurotoxin type A with monoclonal antibodies that map to light-chain epitopes. Eur. J. Biochem..

[227] Facchiano F., Benfenati F., Valtorta F., Luini A. (1993). Covalent modification of synapsin I by a tetanus toxin-activated transglutaminase. J. Biol. Chem..

[228] Facchiano F., Luini A. (1992). Tetanus toxin potently stimulates tissue transglutaminase. A possible mechanism of neurotoxicity. J. Biol. Chem..

[229] Coffield J.A., Considine R.V., Jeyapaul J., Maksymowych A.B., Zhang R.D., Simpson L.L. (1994). The role of transglutaminase in the mechanism of action of tetanus toxin. J. Biol. Chem..

[230] Gobbi M., Frittoli E., Mennini T. (1996). Role of transglutaminase in [3H]5-HT release from synaptosomes and in the inhibitory effect of tetanus toxin. Neurochem. Int..

[231] Fesus L., Piacentini M. (2002). Transglutaminase 2: An enigmatic enzyme with diverse functions. Trends Biochem. Sci..

[232] Lorand L., Graham R.M. (2003). Transglutaminases: Crosslinking enzymes with pleiotropic functions. Nat. Rev. Mol. Cell Biol..

[233] Maggio N., Sellitti S., Capano C.P., Papa M. (2001). Tissue-transglutaminase in rat and human brain: light and electron immunocytochemical analysis and *in situ* hybridization study. Brain Res. Bull..

[234] Walther D.J., Peter J.U., Winter S., Holtje M., Paulmann N., Grohmann M., Vowinckel J., Alamo-Bethencourt V., Wilhelm C.S., Ahnert-Hilger G., Bader M. (2003). Serotonylation of small GTPases is a signal transduction pathway that triggers platelet alpha-granule release. Cell.

[235] Driscoll H.K., Adkins C.D., Chertow T.E., Cordle M.B., Matthews K.A., Chertow B.S. (1997). Vitamin A stimulation of insulin secretion: effects on transglutaminase mRNA and activity using rat islets and insulin-secreting cells. Pancreas.

[236] Pastuszko A., Wilson D.F., Erecinska M. (1986). A role for transglutaminase in neurotransmitter release by rat brain synaptosomes. J. Neurochem..

[237] Humeau Y., Doussau F., Vittello F., Greengard P., Benfenati F., Poulain B. (2001). Synapsin controls both reserve and releasable synaptic vesicle pools during neuronal activity and short-term plasticity in *Aplysia*. J. Neurosci..

[238] Baldelli P., Fassio A., Valtorta F., Benfenati F. (2007). Lack of synapsin I reduces the readily releasable pool of synaptic vesicles at central inhibitory synapses. J. Neurosci..

[239] Presek P., Jessen S., Dreyer F., Jarvie P.E., Findik D., Dunkley P.R. (1992). Tetanus toxin inhibits depolarization-stimulated protein phosphorylation in rat cortical synaptosomes: Effect on synapsin I phosphorylation and translocation. J. Neurochem..

[240] Dayanithi G., Stecher B., Höhne-Zell B., Yamasaki S., Binz T., Weller U., Niemann H., Gratzl M. (1994). Exploring the functional domain and the target of the tetanus toxin light chain in neurophysial terminals. Neuroscience.

[241] DasGupta B.R., Tepp W. (1993). Protease activity of botulinum neurotoxin type E and its light chain: Cleavage of actin. Biochem. Biophys. Res. Commun..

[242] Marxen P., Bigalke H. (1991). Tetanus and botulinum A toxins inhibit stimulated F-actin rearrangement in chromaffin cells. Neuroreport.

[243] Eisel U., Reynolds K., Riddick M., Zimmer A., Niemann H., Zimmer A. (1993). Tetanus toxin light chain expression in Sertoli cells of transgenic mice causes alterations of the actin cytoskeleton and disrupts spermatogenesis. EMBO J..

[244] Ishida H., Zhang X., Erickson K., Ray P. (2004). Botulinum toxin type A targets RhoB to inhibit lysophosphatidic acid-stimulated actin reorganization and acetylcholine release in nerve growth factor-treated PC12 cells. J. Pharmacol. Exp. Ther..

[245] Nevins A.K., Thurmond D.C. (2005). A Direct interaction between Cdc42 and vesicle-associated membrane protein 2 Regulates SNARE-dependent insulin exocytosis. J. Biol. Chem..

[246] Aguilera J., Yavin E. (1990). *In vivo* translocation and down-regulation of protein kinase C following intraventricular administration of tetanus toxin. J. Neurochem..

[247] Gil C., Ruiz-Meana M., Alava M., Yavin E., Aguilera J. (1998). Tetanus toxin enhances protein kinase C activity translocation and increases polyphosphoinositide hydrolysis in rat cerebral cortex preparations. J. Neurochem..

[248] Gil C., Chaib-Oukadour I., Pelliccioni P., Aguilera J. (2000). Activation of signal transduction pathways involving trkA, PLCgamma-1, PKC isoforms and ERK-1/2 by tetanus toxin. FEBS Lett..

[249] Gil C., Chaib-Oukadour I., Aguilera J. (2003). C-terminal fragment of tetanus toxin heavy chain activates Akt and MEK/ERK signalling pathways in a Trk receptor-dependent manner in cultured cortical neurons. Biochem. J..

[250] Chaib-Oukadour I., Gil C., Aguilera J. (2004). The C-terminal domain of the heavy chain of tetanus toxin rescues cerebellar granule neurones from apoptotic death: Involvement of phosphatidylinositol 3-kinase and mitogen-activated protein kinase pathways. J. Neurochem..

[251] Chaib-Oukadour I., Gil C., Rodriguez-Alvarez J., Ortega A., Aguilera J. (2009). Tetanus toxin H(C) fragment reduces neuronal MPP+ toxicity. Mol. Cell Neurosci..

[252] Mendieta L., Venegas B., Moreno N., Patricio A., Martinez I., Aguilera J., Limon I.D. (2009). The carboxyl-terminal domain of the heavy chain of tetanus toxin prevents dopaminergic degeneration and improves motor behavior in rats with striatal MPP(+)-lesions. Neurosci. Res..

[253] Chaib-Oukadour I., Gil C., Aguilera J. (2004). The C-terminal domain of the heavy chain of tetanus toxin rescues cerebellar granule neurones from apoptotic death: Involvement of phosphatidylinositol 3-kinase and mitogen-activated protein kinase pathways. J. Neurochem..

[254] Jank T., Aktories K. (2008). Structure and mode of action of clostridial glucosylating toxins: The ABCD model. Trends Microbiol..

[255] Just I., Selzer J., Wilm M., von Eichel-Streiber C., Mann M., Aktories K. (1995). Glucosylation of Rho proteins by *Clostridium difficile* toxin B. Nature (London).

[256] Just I., Wilm M., Selzer J., Rex G., von Eichel-Streiber C., Mann M., Aktories K. (1995). The enterotoxin from *Clostridium difficile* (ToxA) monoglucosylates the Rho proteins. J. Biol. Chem..

[257] Popoff M.R., Chaves-Olarte E., Lemichez E., Von Eichel-Streiber C., Thelestam M., Chardin P., Cussac D., Antonny B., Chavrier P., Flatau G., Giry M., de Gunzburg J., Boquet P. (1996). Ras, Rap, and rac small GTP-binding proteins are targets for *Clostridium sordellii* lethal toxin glucosylation. J. Biol. Chem..

[258] Hermann C., Ahmadian M.R., Hofmann F., Just I. (1998). Functional consequences of monoglucosylation of Ha-Ras at effector domain amino acid threonine 35. J. Biol. Chem..

[259] Vetter I.R., Hofmann F., Wohlgemuth S., Hermann C., Just I. (2000). Structural consequences of monoglucosylation of Ha-Ras by *Clostridium sordellii* lethal toxin. J. Mol. Biol..

[260] Popoff M.R., Bouvet P. (2009). Clostridial toxins. Future Microbiol..

[261] Aktories K., Just I. (2005). Clostridial Rho-inhibiting protein toxins. Curr. Top Microbiol. Immunol..

[262] Vogelsgesang M., Pautsch A., Aktories K. (2007). C3 exoenzymes, novel insights into structure and action of Rho-ADP-ribosylating toxins. Naunyn Schmiedebergs Arch. Pharmacol..

[263] Djouder N., Aneiros E., Cavalie A., Aktories K. (2003). Effects of large clostridial cytotoxins on activation of RBL 2H3-hm1 mast cells indicate common and different roles of Rac in FcepsilonRI and M1-receptor signaling. J. Pharmacol. Exp. Ther..

[264] Gasman S., Chasserot-Golaz S., Popoff M.R., Aunis D., Bader M.F. (1999). Involvement of Rho GTPases in calcium-regulated exocytosis from adrenal chromaffin cells. J. Cell Sci..

[265] Kowluru A., Li G., Rabaglia M.E., Segu V.B., Hofmann F., Aktories K., Metz S.A. (1997). Evidence for differential roles of the Rho subfamily of GTP-binding proteins in glucose- and calcium-induced insulin secretion from pancreatic beta cells. Biochem. Pharmacol..

[266] Prepens U., Just I., von Eichel-Streiber C., Aktories K. (1996). Inhibition of Fc epsilon-RI-mediated activation of rat basophilic leukemia cells by Clostridium difficile toxin B (monoglucosyltransferase). J. Biol. Chem..

[267] Barbier J., Popoff M.R., Molgo J. (2004). Degeneration and regeneration of murine skeletal neuromuscular junctions after intramuscular injection with a sublethal dose of *Clostridium sordellii* lethal toxin. Infect. Immun..

[268] Geny B., Khum H., Fitting C., Zarantonelli L., Mazuet C., Cayet N., Szatanik M., Prevost M.C., Cavaillon J.M., Huerre M., Popoff M.R. (2007). *Clostridium sordellii* lethal toxin kills mice by inducing a major increase in lung vascular permeability. Am. J. Pathol..

[269] Pothoulakis C., Castagliuolo I., LaMont J.T. (1998). Nerves and intestinal mast cells modulate responses to enterotoxins. News Physiol. Sci..

[270] Djouder N., Prepens U., Aktories K., Cavalie A. (2000). Inhibition of calcium release-activated calcium current by Rac/Cdc42-inactivating clostridial cytotoxins in RBL cells. J. Biol. Chem..

[271] Short B., Barr F.A. (2002). Membrane traffic: Exocyst III--makes a family. Curr. Biol..

[272] Ben El Hadj N., Popoff M.R., Marvaud J.C., Payrastre B., Boquet P., Geny B. (1999). G-protein-stimulated phospholipase D activity is inhibited by lethal toxin from *Clostridium sordellii* in HL-60 cells. J. Biol. Chem..

[273] Hammond K., Caputo G.A., London E. (2002). Interaction of the membrane-inserted diphtheria toxin T domain with peptides and its possible implications for chaperone-like T domain behavior. Biochemistry.

[274] Meyer D.K., Olenik C., Hofmann F., Barth H., Leemhuis J., Brunig I., Aktories K., Norenberg W. (2000). Regulation of somatodendritic GABAA receptor channels in rat hippocampal neurons: Evidence for a role of the small GTPase Rac1. J. Neurosci..

[275] Murray H.J., O'Connor J.J. (2004). A role for monomeric G-proteins in synaptic plasticity in the rat dentate gyrus *in votro*. Brain Res..

[276] Triller A., Choquet D. (2003). Synaptic structure and diffusion dynamics of synaptic receptors. Biol. Cell.

[277] Linseman D.A., Laessig T., Meintzer M.K., McClure M., Barth H., Aktories K., Heidenreich K.A. (2001). An essential role for Rac/Cdc42 GTPases in cerebellar granule neuron survival. J. Biol. Chem..

[278] Marriott H.M., Mitchell T.J., Dockrell D.H. (2008). Pneumolysin: A double-edged sword during the host-pathogen interaction. Curr. Mol. Med..

[279] Rossjohn J., Gilbert R.J., Crane D., Morgan P.J., Mitchell T.J., Rowe A.J., Andrew P.W., Paton J.C., Tweten R.K., Parker M.W. (1998). The molecular mechanism of pneumolysin, a virulence factor from Streptococcus pneumoniae. J. Mol. Biol..

[280] Soltani C.E., Hotze E.M., Johnson A.E., Tweten R.K. (2007). Structural elements of the cholesterol-dependent cytolysins that are responsible for their cholesterol-sensitive membrane interactions. Proc. Natl. Acad. Sci. USA.

[281] Mitchell T.J., Alouf J.E., Popoff M.R. (2006). Pneumolysin: Structure, function, and role in disease. The Sourcebook of Bacterial Protein Toxins.

[282] Soltani C.E., Hotze E.M., Johnson A.E., Tweten R.K. (2007). Specific protein-membrane contacts are required for prepore and pore assembly by a cholesterol-dependent cytolysin. J. Biol. Chem..

[283] Shepard L., Shatursky O., Johnson A., Tweten R. (2000). The mechanism of pore assembly for a cholesterol-dependent cytolysin: formation of a large prepore complex precedes the insertion of hte transmembrane b-hairpins. Biochemistry.

[284] Dang T.X., Hotze E.M., Rouiller I., Tweten R.K., Wilson-Kubalek E.M. (2005). Prepore to pore transition of a cholesterol-dependent cytolysin visualized by electron microscopy. J. Struct. Biol..

[285] Ramachandran R., Tweten R.K., Johnson A.E. (2004). Membrane-dependent conformational changes initiate cholesterol-dependent cytolysin oligomerization and intersubunit β-strand alignment. Nat. Struct. Mol. Biol..

[286] Heuck A.P., Savva C.G., Holzenburg A., Johnson A.E. (2007). Conformational changes that effect oligomerization and initiate pore formation are triggered throughout perfringolysin O upon binding to cholesterol. J. Biol. Chem..

[287] Rossjohn J., Polekhina G., Feil S.C., Morton C.J., Tweten R.K., Parker M.W. (2007). Structures of perfringolysin O suggest a pathway for activation of cholesterol-dependent cytolysins. J. Mol. Biol..

[288] Ramachandran R., Heuck A.P., Tweten R.K., Johnson A.E. (2002). Structural insights into the membrane-anchoring mechanism of a cholesterol-dependent cytolysin. Nat. Struct. Biol..

[289] Heuck A.P., Hotze E.M., Tweten R.K., Johnson A.E. (2000). Mechanism of membrane insertion of a multimeric beta-barrel protein: Perfringolysin O creates a pore using ordered and coupled conformational changes. Mol. Cell..

[290] Heuck A.P., Tweten R.K., Johnson A.E. (2001). β-barrel pore-forming toxins: Intriguing dimorphic proteins. Biochemistry.

[291] Shatursky O., Heuck A., Shepard L., Rossjhon J., Parker M., Johnson A., Tweten R. (1999). The mechanism of membrane insertion of a cholesterol-dependent cytolysin: A novel paradigm for pore-forming toxins. Cell.

[292] Hotze E.M., Heuck A.P., Czajkowsky D.M., Shao Z., Johnson A.E., Tweten R.K. (2002). Monomer-monomer interactions drive the prepore to pore conversion of a β-barrel-forming cholesterol-dependent cytolysin. J. Biol. Chem..

[293] Rossjohn J., Feil S.C., McKinstry W.J., Tweten R.K., Parker M.W. (1997). Structure of a cholesterol-binding thiol-activated cytolysin and a model of its membrane form. Cell.

[294] Czajkowsky D.M., Hotze E.M., Shao Z., Tweten R.K. (2004). Vertical collapse of a cytolysin prepore moves its transmembrane beta-hairpins to the membrane. Embo J..

[295] Hirst R.A., Kadioglu A., O'Callaghan C., Andrew P.W. (2004). The role of pneumolysin in pneumococcal pneumonia and meningitis. Clin. Exp. Immunol..

[296] Braun J.S., Sublett J.E., Freyer D., Mitchell T.J., Cleveland J.L., Tuomanen E.I., Weber J.R. (2002). Pneumococcal pneumolysin and H(2)O(2) mediate brain cell apoptosis during meningitis. J. Clin. Invest..

[297] Braun J.S., Hoffmann O., Schickhaus M., Freyer D., Dagand E., Bermpohl D., Mitchell T.J., Bechmann I., Weber J.R. (2007). Pneumolysin causes neuronal cell death through mitochondrial damage. Infect. Immun..

[298] Stringaris A.K., Geisenhainer J., Bergmann F., Balshusemann C., Lee U., Zysk G., Mitchell T.J., Keller B.U., Kuhnt U., Gerber J., Spreer A., Bahr M., Michel U., Nau R. (2002). Neurotoxicity of pneumolysin, a major pneumococcal virulence factor, involves calcium influx and depends on activation of p38 mitogen-activated protein kinase. Neurobiol. Dis..

[299] Goldstein J., Morris W.E., Loidl C.F., Tironi-Farinatti C., McClane B.A., Uzal F.A., Fernandez Miyakawa M.E. (2009). Clostridium perfringens epsilon toxin increases the small intestinal permeability in mice and rats. PLoS One.

[300] Losada-Eaton D.M., Uzal F.A., Fernandez Miyakawa M.E. (2008). Clostridium perfringens epsilon toxin is absorbed from different intestinal segments of mice. Toxicon.

[301] Finnie J.W. (2003). Pathogenesis of brain damage produced in sheep by Clostridium perfringens type D epsilon toxin: A review. Aust. Vet J..

[302] Payne D., Williamson E.D., Titball R.W. (1997). The *Clostridium perfringens* epsilon-toxin. Rev. Med. Microbiol..

[303] Nagahama M., Sakurai J. (1991). Distribution of labeled *Clostridium perfringens* epsilon toxin in mice. Toxicon.

[304] Nagahama M., Sakurai J. (1992). High-affinity binding of *Clostridium perfringens* epsilon-toxin to rat brain. Infect. Immun..

[305] Zhu C., Ghabriel M.N., Blumbergs P.C., Reilly P.L., Manavis J., Youssef J., Hatami S., Finnie J.W. (2001). *Clostridium perfringens* prototoxin-induced alteration of endothelial barrier antigen (EBA) immunoreactivity at the blood brain barrier (BBB). Exp. Neurol..

[306] Buxton D. (1978). The use of an imunoperoxidase technique to investigate by light and electron microscopy the sites of binding of *Clostridium welchii* type D e-toxin in mice. J. Med. Microbiol..

[307] Finnie J.W., Blumbergs P.C., Manavis J. (1999). Neuronal damage produced in rat brains by *Clostridium perfringens* type D epsilon-toxin. J. Comp. Path..

[308] Miyamoto O., Minami J., Toyoshima T., Nakamura T., Masada T., Nagao S., Negi T., Itano T., Okabe A. (1998). Neurotoxicity of *Clostridium perfringens* epsilon-toxin for the rat hipocampus *via* glutamanergic system. Infect. Immun..

[309] Miyamoto O., Sumitami K., Nakamura T., Yamagani S., Miyatal S., Itano T., Negi T., Okabe A. (2000). *Clostridium perfringens* epsilon toxin causes excessive release of glutamate in the mouse hippocampus. FEMS Microbiol. Lett..

[310] Cole A.R., Gibert M., Popoff M.R., Moss D.S., Titball R.W., Basak A. (2004). *Clostridium perfringens* ε-toxin shows structural similarity to the pore-forming toxin aerolysin. Nat. Struct. Mol. Biol..

[311] Knapp O., Maier E., Benz R., Geny B., Popoff M.R. (2009). Identification of the channel-forming domain of Clostridium perfringens Epsilon-toxin (ETX). Biochim. Biophys. Acta.

[312] Cole A., Duchesnes C., Mainil J., Popoff M.R., Titball R. (2003). Structural studies on epsilon toxin from *Clostridium perfringens*. Protein Toxins of the Genus Clostridium and Vaccination.

[313] Miyata S., Minami J., Tamai E., Matsushita O., Shimamoto S., Okabe A. (2002). *Clostridium perfringens* ε-toxin forms a heptameric pore within the detergent-insoluble microdomains of Madin-Darby Canine Kidney Cells and rat synaptosomes. J. Biol. Chem..

[314] Petit L., Gibert M., Gillet D., Laurent-Winter C., Boquet P., Popoff M.R. (1997). *Clostridium perfringens* epsilon-toxin acts on MDCK cells by forming a large membrane complex. J. Bacteriol..

[315] Petit L., Maier E., Gibert M., Popoff M.R., Benz R. (2001). Clostridium perfringens epsilon-toxin induces a rapid change in cell membrane permeability to ions and forms channels in artificial lipid bilayers. J. Biol. Chem..

[316] Petit P., Breard J., Montalescol V., Ben El Hadj N., Levade T., Popoff M.R., Geny B. (2003). Lethal toxin from Clostridium sordellii induces apoptotic cell death by disruption of mitochondrial homeostasis in HL-60 cells. Cell. Miccrobiol..

[317] Miyata S., Matsushita O., Minami J., Katayama S., Shimamoto S., Okabe A. (2001). Cleavage of C-terminal peptide is essential for heptamerization of *Clostridium perfringens* ε-toxin in the synaptosomal membrane. J. Biol. Chem..

[318] Petit L., Gibert M., Gourch A., Bens M., Vandewalle A., Popoff M.R. (2003). *Clostridium perfringens* Epsilon Toxin rapidly decreases membrane barrier permeability of polarized MDCK Cells. Cell. Microbiol..

[319] Chassin C., Bens M., de Barry J., Courjaret R., Bossu J.L., Cluzeaud F., Ben Mkaddem S., Gibert M., Poulain B., Popoff M.R., Vandewalle A. (2007). Pore-forming epsilon toxin causes membrane permeabilization and rapid ATP depletion-mediated cell death in renal collecting duct cells. Am. J. Physiol. Renal. Physiol..

[320] Farthing M.J. (2000). Enterotoxins and the enteric nervous system--a fatal attraction. Int. J. Med. Microbiol..

[321] Farthing M.J.G., Casburn-Jones A., Banks M.R. (2004). Enterotoxins, enteric nerves, and intestinal secretion. Curr. Gastroenterol. Rep..

[322] Hirst T.R., D'Souza J.M., Alouf J.E., Popoff M.R. (2006). *Vibrio cholerae* and *Escherichia coli* thermolabile enterotoxin. The Sourcebook of Bacterial Protein Toxins.

[323] Holmes R.K., Jobling M.G., Conell T.D., Moss J., Iglewski B., Vaughan M., Tu A.T. (1995). Cholera toxin and related enterotoxins of Gram-negative bacteria. Bacterial Toxins and Virulence Factors in Disease.

[324] de Haan L., Hirst T.R. (2004). Cholera toxin: A paradigm for multi-functional engagement of cellular mechanisms. Mol. Membr. Biol..

[325] Nichols B.J. (2002). A distinct class of endosome mediates clathrin-independent endocytosis to the Golgi complex. Nat. Cell Biol..

[326] Fujinaga Y., Wolf A.A., Rodighiero C., Wheeler H.E., Tsai B., Allen L., Jobling M.G., Rapoport T., Holmes R.K., Lencer W.I. (2003). Gangliosides that associate with lipid rafts mediate transport of cholera and related toxins from the plasma membrane to endoplasmic reticulum. Mol. Biol. Cell.

[327] Johannes L., Tenza D., Antony C., Goud B. (1997). Retrograde transport of KDEL-bearing B-fragment of Shiga toxin. J. Biol. Chem..

[328] Lundgren O. (1998). 5-Hydroxytryptamine, enterotoxins, and intestinal fluid secretion. Gastroenterology.

[329] Turvill J.L., Mourad F.H., Farthing M.J. (1998). Crucial role for 5-HT in cholera toxin but not Escherichia coli heat-labile enterotoxin-intestinal secretion in rats. Gastroenterology.

[330] Mourad F.H., O'Donnell L.J., Dias J.A., Ogutu E., Andre E.A., Turvill J.L., Farthing M.J. (1995). Role of 5-hydroxytryptamine type 3 receptors in rat intestinal fluid and electrolyte secretion induced by cholera and Escherichia coli enterotoxins. Gut.

[331] Bearcroft C.P., Perrett D., Farthing M.J. (1996). 5-hydroxytryptamine release into human jejunum by cholera toxin. Gut.

[332] Nilsson O., Cassuto J., Larsson P.A., Jodal M., Lidberg P., Ahlman H., Dahlstrom A., Lundgren O. (1983). 5-Hydroxytryptamine and cholera secretion: A histochemical and physiological study in cats. Gut.

[333] Beubler E., Horina G. (1990). 5-HT2 and 5-HT3 receptor subtypes mediate cholera toxin-induced intestinal fluid secretion in the rat. Gastroenterology.

[334] Buchheit K.H. (1989). Inhibition of cholera toxin-induced intestinal secretion by the 5-HT3 receptor antagonist ICS 205–930. Naunyn Schmiedebergs Arch. Pharmacol..

[335] Beattie D.T., Smith J.A. (2008). Serotonin pharmacology in the gastrointestinal tract: A review. Naunyn Schmiedebergs Arch. Pharmacol..

[336] Cooke H.J. (2000). Neurotransmitters in neuronal reflexes regulating intestinal secretion. Ann. NY Acad. Sci..

[337] Lundgren O. (2002). Enteric nerves and diarrhoea. Pharmacol. Toxicol..

[338] Turvill J.L., Connor P., Farthing M.J. (2000). Neurokinin 1 and 2 receptors mediate cholera toxin secretion in rat jejunum. Gastroenterology.

[339] Castagliuolo I., Lamont J.T., Letourneau R., Kelly C., O'Keane J.C., Jaffer A., Theoharides T.C., Pothoulakis C. (1994). Neuronal involvement in the intestinal effects of *Clostridium difficile* Toxin A and *Vibrio cholerae* enterotoxin in rat ileum. Gastroenterology.

[340] Pothoulakis C., Lamont J.T. (2001). Microbes and microbial toxins: paradigms for microbial-mucosa interactions II. The integrated response of the intestine to *Clostridium difficile* toxins. Am. J. Physiol. Gastrointest. Liver Physiol..

[341] Castagliuolo I., Riegler M., Pasha A., Nikulasson S., Lu B., Gerard C., Gerard N.P., Pothoulakis C. (1998). Neurokinin-1 (NK-1) receptor is required in *Clostridium difficile*-induced enteritis. J. Clin. Invest..

[342] Castagliuolo I., Wang C.C., Valenick L., Pasha A., Nikulasson S., Carraway R.E., Pothoulakis C. (1999). Neurotensin is a proinflammatory peptide in colonic inflammation. J. Clin. Invest..

[343] Xia Y., Hu H.Z., Liu S., Pothoulakis C., Wood J.D. (2000). *Clostridium difficile* toxin A excites enteric neurones and suppresses sympathetic neurotransmision in the guinea pig. Gut.

[344] McClane B.A., Alouf J.E., Popoff M.R. (2006). *Clostridium perfringens* enterotoxin. The Cmprehensive Sourcebook of Bacterial Protein Toxins.

[345] Senda T., Sugimoto N., Horiguchi Y., Matsuda M. (1995). The enterotoxin of Clostridium perfringens type A binds to the presynaptic nerve endings in neuromuscular junctions of mouse phrenic nerve-diaphragm. Toxicon.

[346] Rolfe V.E., Levin R.J. (1999). Vagotomy inhibits the jejunal fluid secretion activated by luminal ileal Escherichia coli STa in the rat *in vivo*. Gut.

[347] Mourad F.H., Nassar C.F. (2000). Effect of vasoactive intestinal polypeptide (VIP) antagonism on rat jejunal fluid and electrolyte secretion induced by cholera and Escherichia coli enterotoxins. Gut.

[348] Rolfe V., Levin R.J. (1994). Enterotoxin Escherichia coli STa activates a nitric oxide-dependent myenteric plexus secretory reflex in the rat ileum. J. Physiol..

[349] Eklund S., Jodal M., Lundgren O. (1985). The enteric nervous system participates in the secretory response to the heat stable enterotoxins of Escherichia coli in rats and cats. Neuroscience.

[350] Stenfors Arnesen L.P., Fagerlund A., Granum P.E. (2008). From soil to gut: Bacillus cereus and its food poisoning toxins. FEMS Microbiol. Rev..

[351] Toh M., Moffitt M.C., Henrichsen L., Raftery M., Barrow K., Cox J.M., Marquis C.P., Neilan B.A. (2004). Cereulide, the emetic toxin of Bacillus cereus, is putatively a product of nonribosomal peptide synthesis. J. Appl. Microbiol..

[352] Agata N., Ohta M., Mori M., Isobe M. (1995). A novel dodecadepsipeptide, cereulide, is an emetic toxin of Bacillus cereus. FEMS Microbiol. Lett..

[353] Horwood P.F., Burgess G.W., Oakey H.J. (2004). Evidence for non-ribosomal peptide synthetase production of cereulide (the emetic toxin) in Bacillus cereus. FEMS Microbiol. Lett..

[354] Agata N., Ohta M., Mori M. (1996). Production of an emetic toxin, cereulide, is associated with a specific class of Bacillus cereus. Curr. Microbiol..

[355] Shinagawa K., Konuma H., Sekita H., Sugii S. (1995). Emesis of rhesus monkeys induced by intragastric administration with the HEp-2 vacuolation factor (cereulide) produced by Bacillus cereus. FEMS Microbiol. Lett..

[356] Agata N., Mori M., Ohta M., Suwan S., Ohtani I., Isobe M. (1994). A novel dodecadepsipeptide, cereulide, isolated from Bacillus cereus causes vacuole formation in HEp-2 cells. FEMS Microbiol. Lett..

[357] Yokoyama K., Ito M., Agata N., Isobe M., Shibayama K., Horii T., Ohta M. (1999). Pathological effect of synthetic cereulide, an emetic toxin of Bacillus cereus, is reversible in mice. FEMS Immunol. Med. Microbiol..

[358] Virtanen S.M., Roivainen M., Andersson M.A., Ylipaasto P., Hoornstra D., Mikkola R., Salkinoja-Salonen M.S. (2008). *In vitro* toxicity of cereulide on porcine pancreatic Langerhans islets. Toxicon.

[359] Andersson M.A., Hakulinen P., Honkalampi-Hamalainen U., Hoornstra D., Lhuguenot J.C., Maki-Paakkanen J., Savolainen M., Severin I., Stammati A.L., Turco L., Weber A., von Wright A., Zucco F., Salkinoja-Salonen M. (2007). Toxicological profile of cereulide, the Bacillus cereus emetic toxin, in functional assays with human, animal and bacterial cells. Toxicon.

[360] Mikkola R., Saris N.E., Grigoriev P.A., Andersson M.A., Salkinoja-Salonen M.S. (1999). Ionophoretic properties and mitochondrial effects of cereulide: the emetic toxin of B. cereus. Eur. J. Biochem..

[361] Teplova V.V., Mikkola R., Tonshin A.A., Saris N.E., Salkinoja-Salonen M.S. (2006). The higher toxicity of cereulide relative to valinomycin is due to its higher affinity for potassium at physiological plasma concentration. Toxicol. Appl. Pharmacol..

[362] Saris N.E., Andersson M.A., Mikkola R., Andersson L.C., Teplova V.V., Grigoriev P.A., Salkinoja-Salonen M.S. (2009). Microbial toxin's effect on mitochondrial survival by increasing K^+^ uptake. Toxicol. Ind. Health..

[363] Krakauer T., Stiles B.G. (2003). Staphylococcal enterotoxins, toxic-shock syndrome toxin-1, and streptococcal pyrogenic exotoxins: Some basic biology of bacterial superantigens. Rec. Res. Dev. Infect. Immun..

[364] Jett M., Brinkley W., Neill R., Gemski P., Hunt R. (1990). Staphylococcus aureus enterotoxin B challenge of monkeys: Correlation of plasma levels of arachidonic acid cascade products with occurrence of illness. Infect. Immun..

[365] Alber G., Scheuber P.H., Reck B., Sailer-Kramer B., Hartmann A., Hammer D.K. (1989). Role of substance P in immediate-type skin reactions induced by staphylococcal enterotoxin B in unsensitized monkeys. J. Allergy Clin. Immunol..

[366] Tiegs G., Bang R., Neuhuber W.L. (1999). Requirement of peptidergic sensory innervation for disease activity in murine models of immune hepatitis and protection by beta-adrenergic stimulation. J. Neuroimmunol..

[367] Wang X., Wang B.R., Zhang X.J., Duan X.L., Guo X., Ju G. (2004). Fos expression in the rat brain after intraperitoneal injection of Staphylococcus enterotoxin B and the effect of vagotomy. Neurochem. Res..

[368] Hu D.L., Zhu G., Mori F., Omoe K., Okada M., Wakabayashi K., Kaneko S., Shinagawa K., Nakane A. (2007). Staphylococcal enterotoxin induces emesis through increasing serotonin release in intestine and it is downregulated by cannabinoid receptor 1. Cell Microbiol..

